# Programmed cell death 10 promotes metastasis and epithelial-mesenchymal transition of hepatocellular carcinoma via PP2Ac-mediated YAP activation

**DOI:** 10.1038/s41419-021-04139-z

**Published:** 2021-09-14

**Authors:** Bo Sun, Fang-Jing Zhong, Cong Xu, Yi-Ming Li, Yan-Rong Zhao, Mo-Mo Cao, Lian-Yue Yang

**Affiliations:** 1 Liver Cancer Laboratory, Xiangya Hospital, Central South University, Changsha, 410008 Hunan China; 2 Department of Surgery, Xiangya Hospital, Central South University, Changsha, 410008 Hunan China

**Keywords:** Metastasis, Oncogenes, Prognostic markers

## Abstract

Tumour metastasis is the main cause of postoperative tumour recurrence and mortality in patients with hepatocellular carcinoma (HCC), but the underlying mechanism remains unclear. Accumulating evidence has demonstrated that programmed cell death 10 (PDCD10) plays an important role in many biological processes. However, the role of PDCD10 in HCC progression is still elusive. In this study, we aimed to explore the clinical significance and molecular function of PDCD10 in HCC. PDCD10 is significantly upregulated in HCC, which also correlates with aggressive clinicopathological characteristics and predicts poor prognosis of HCC patients after liver resection. High PDCD10 expression promotes HCC cell proliferation, migration, and invasion in vitro and tumour growth, metastasis in vivo. In addition, PDCD10 could facilitate epithelial-to-mesenchymal transition (EMT) of HCC cells. In terms of the mechanism, PDCD10 directly binds to the catalytic subunit of protein phosphatase 2A (PP2Ac) and increases its enzymatic activity, leading to the interaction of YAP and dephosphorylation of the YAP protein. This interaction contributes to YAP nuclear translocation and transcriptional activation. PP2Ac is necessary for PDCD10-mediated HCC progression. Knocking down PP2Ac abolished the tumour-promoting role of PDCD10 in the migration, invasion and EMT of HCC. Moreover, a PP2Ac inhibitor (LB100) could restrict tumour growth and metastasis of HCC with high PDCD10 expression. Collectively, PDCD10 promotes EMT and the progression of HCC by interacting with PP2Ac to promote YAP activation, which provides new insight into the mechanism of cancer metastasis. PDCD10 may be a potential prognostic biomarker and therapeutic target for HCC.

## Introduction

Hepatocellular carcinoma (HCC) is predicted to be the sixth most commonly diagnosed cancer and the fourth leading cause of cancer death worldwide [1]. Although the diagnosis and treatment of HCC have evolved considerably over the past decade, metastasis-associated recurrence is still the major cause of poor prognosis for HCC patients [2, 3]. However, the precise mechanisms underlying the invasion and metastasis of HCC remain largely unknown and there is still a lack of effective treatment for HCC metastasis. Therefore, it is urgent to further investigate the mechanism of HCC invasion and metastasis.

Programmed cell death 10 (PDCD10) was originally discovered in human premyeloid cells to study the genes induced during apoptotic cell death and was found to be highly expressed in the foetal liver [4]. PDCD10 interacts with a collection of signalling, cytoskeletal and adaptor proteins and plays a vital role in a range of cellular processes including cell apoptosis, proliferation, and migration [5]. However, the function of PDCD10 is tissue- and disease-specific. PDCD10 could promote ERM phosphorylation after oxidative stress and has a prosurvival action [6]. PDCD10 expression is also linked to cell death, and loss of PCDC10 has been reported to increase survival and proliferation [7]. Studies from the Zhu group indicate that PDCD10 plays a tumour suppressor role in glioblastoma by inhibiting growth and invasion [8, 9]. More evidence suggests that PDCD10 acts as an oncogene in some epithelial malignant tumours, such as breast cancer [10], ovarian cancer [11], and non-small cell lung cancer [12]. Many public databases, such as Oncomine, the Gene Expression Omnibus (GEO), and The Cancer Genome Atlas (TCGA), show that PDCD10 is significantly upregulated in HCC tissues. Nonetheless, PDCD10 has never been researched in HCC. Here, we systematically studied the clinical significance and functional role of PDCD10 in HCC.

In this study, we found that PDCD10 is frequently upregulated in HCC and closely associated with aggressive clinicopathologic characteristics and poor prognosis of HCC patients after liver resection. Functionally, PDCD10 promotes HCC cell proliferation, migration, and invasion in vitro and growth, and metastasis in vivo and is related to epithelial–mesenchymal transition (EMT). Mechanistically, PDCD10 dephosphorylates YAP to promote its nuclear translocation and transcriptional activity by directly binding with the protein phosphatase type 2 A catalytic subunit (PP2Ac) and increasing PP2Ac enzyme activity. Targeting PP2Ac could abolish the tumour-promoting role of PDCD10 in HCC.

## Results

### PDCD10 is upregulated in HCC tissues and cell lines

**Fig. 1:**
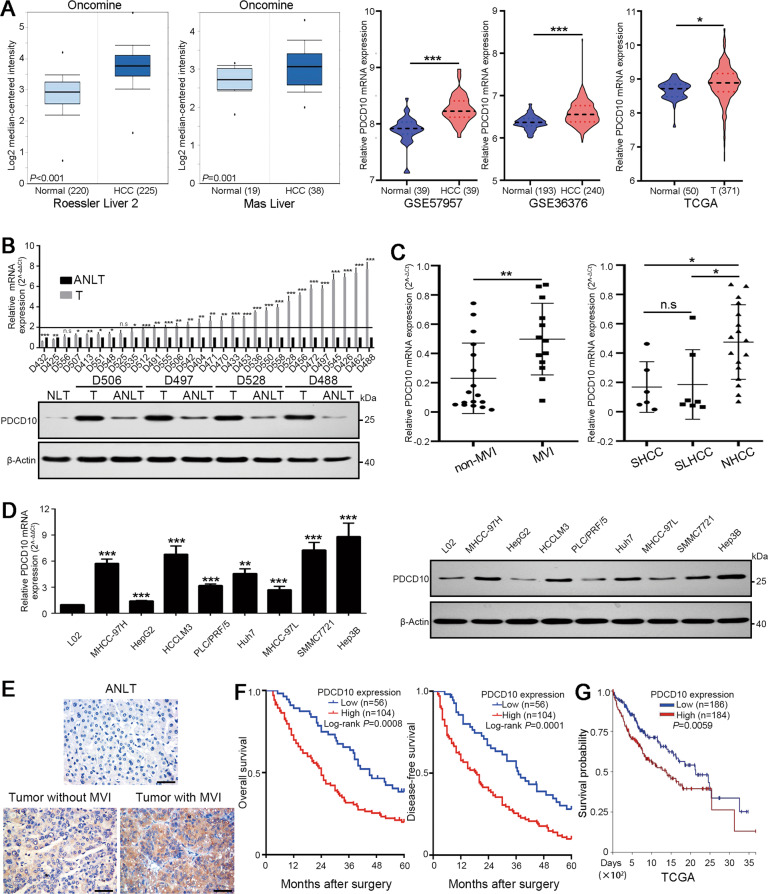
PDCD10 is upregulated in HCC tissues and is associated with poor prognosis of HCC patients. **A** Comparing the levels of PDCD10 mRNA in HCC tissues and normal liver tissues from Oncomine (https://www.oncomine.org/), GEO (GEO accession number: GSE57957 and GSE36376), and TCGA databases. **B** The mRNA and representative protein expression of PDCD10 in fresh frozen ANLTs or NLT and HCC tissues were analysed by qRT-PCR and western blotting. **C** The mRNA expression of PDCD10 in different HCC patient subgroups. **D** The mRNA and protein expression of PDCD10 in L02 and 8 HCC cell lines were analysed by qRT-PCR and western blotting, respectively. **E** Representative IHC images showed the protein expression of PDCD10 in ANLT, and HCC tumour tissues with or without MVI. Scale bars, 50 μm. **F** Kaplan–Meier analysis of overall survival and disease-free survival of HCC patients with high or low PDCD10 expression in the training cohort. **G** Kaplan–Meier analysis of survival probability for HCC patients based on PDCD10 mRNA expression using public data from the TCGA database. ANLT adjacent non-tumourous liver tissue, NLT normal liver tissue, T tumour tissue, MVI microvascular invasion, SHCC small hepatocellular carcinoma, SLHCC solitary large hepatocellular carcinoma, NHCC nodular hepatocellular carcinoma. n.s., no significance; *P < 0.05; **P < 0.01; ***P < 0.001.

Datasets from the Oncomine (https://www.oncomine.org/), GEO (GSE57957, GSE36376), and TCGA databases both showed that PDCD10 mRNA was significantly upregulated in HCC tissues compared with normal liver tissues (Fig. 1A). qRT-PCR results showed that 19 of 30 (63.3%) fresh frozen HCC tissues had significantly upregulated PDCD10 expression compared with their matched adjacent non-tumorous liver tissues (ANLTs) (HCC/ANLT > 2) (Fig. 1B). Western blot analysis confirmed that PDCD10 protein expression was increased in HCC tissues compared with ANLTs and normal liver tissue (NLT) (Fig. 1B). Further analysis showed that HCC with microvascular invasion (MVI) had higher PDCD10 expression than HCC without MVI. Nodular HCC (NHCC) with poor prognosis had higher PDCD10 expression than small HCC (SHCC) and solitary large HCC (SLHCC) with relatively favourable prognosis [13] (Fig. 1C). We further found that HCC cell lines displayed higher mRNA and protein expression of PDCD10 than L02 cells, an immortalized human normal liver cell line (Fig. 1D). Immunohistochemistry (IHC) also revealed that PDCD10 staining was strongest in HCC tumour tissue with MVI, followed by tumour without MVI, and ANLT showed the lowest PDCD10 expression (Fig. 1E). Collectively, these results demonstrated that PDCD10 was upregulated in HCC and may be associated with HCC progression.

### High PDCD10 expression is associated with aggressive clinicopathological characteristics and poor prognosis of HCC patients

To explore the clinical significance of PDCD10 expression in HCC, we first constructed a training and validation cohort (Fig. S1A) and further stratified PDCD10 expression as high and low in the training and validation cohorts according to the IHC scoring mentioned in the “Materials and Methods”. The characteristics of the training and validation cohorts were not significantly different (Table S1). In the training cohort, PDCD10 expression was significantly associated with tumour number, tumour size, MVI, capsular formation, Edmondson-Steiner grade, TNM stage and BCLC stage (all with *P* < 0.05, Table 1). Similar results were also confirmed in the validation cohort (Table 1). Kaplan–Meier survival analysis showed that HCC patients with high PDCD10 expression had significantly shorter overall survival (OS) and disease-free survival (DFS) than those with low PDCD10 expression in the training (Fig. 1F) and validation (Fig. S1B) cohorts. In addition, by analysing survival data from TCGA, we found that HCC patients with high PDCD10 expression had a significantly lower survival probability (Fig. 1G). Univariate and multivariate analyses revealed that high PDCD10 expression was an independent risk factor for both OS and DFS of HCC patients in the training and validation cohorts (Table S2, S3). In summary, these results demonstrated that high PDCD10 expression was correlated with poor prognosis of HCC patients and could serve as a valuable independent prognostic biomarker.

**Table 1:** The association between PDCD10 expression and clinicopathological characteristics of HCC patients in training and validation cohort.

Clinicopathological Variables	Training Cohort				Validation Cohort			
	*n*	PDCD10 Expression		*P* Value	*n*	PDCD10 Expression		*P* Value
		Low	High			Low	High	
*Gender*								
Female	42	17	25		34	12	22	
Male	118	39	79	0.386	106	33	73	0.651
Age (years)								
<60	102	41	61		85	24	61	
≥60	58	15	43	0.068	55	21	34	0.218
*HBsAg*								
Negative	24	6	18		23	10	13	
Positive	136	50	86	0.265	117	35	82	0.203
Liver cirrhosis								
Absence	48	16	32		40	16	24	
Presence	112	40	72	0.772	100	29	71	0.208
AFP (ng/L)								
<20	66	26	40		52	19	33	
≥20	94	30	64	0.329	88	26	62	0.392
*Child-Pugh*								
A	120	45	75		96	28	68	
B	40	11	29	0.251	44	17	27	0.265
*Tumour number*								
Solitary	68	32	36		64	26	38	
Multiple	92	24	68	**0.006**	76	19	57	**0.049**
Tumor size								
≤5 cm	51	24	27		52	24	28	
>5 cm	109	32	77	**0.029**	88	21	67	**0.006**
*Microvascular invasion*								
Absence	87	40	47		82	33	49	
Presence	73	16	57	**0.001**	58	12	46	**0.015**
*Capsular formation*								
Absence	86	24	62		65	17	48	
Presence	74	32	42	**0.043**	75	28	47	0.158
*Edmondson-Steiner grade*								
I–II	86	38	48		71	31	40	
III–IV	74	18	56	**0.009**	69	14	55	**0.003**
TNM stage								
I	65	33	32		58	26	32	
II–III	95	23	72	**0.001**	82	19	63	**0.007**
*BCLC stage*								
0–A	70	32	38		64	28	36	
B–C	90	24	66	**0.012**	76	17	59	**0.007**

*AFP* alpha-fetoprotein, *HBsAg* hepatitis B surface antigen, *TNM* tumor node metastasis, *BCLC* Barcelona Clinic Liver Cancer.

Bold values identify statistical significance (*p* < 0.05).

### PDCD10 promotes HCC cell proliferation, migration, and invasion in vitro and growth, and metastasis in vivo

**Fig. 2:**
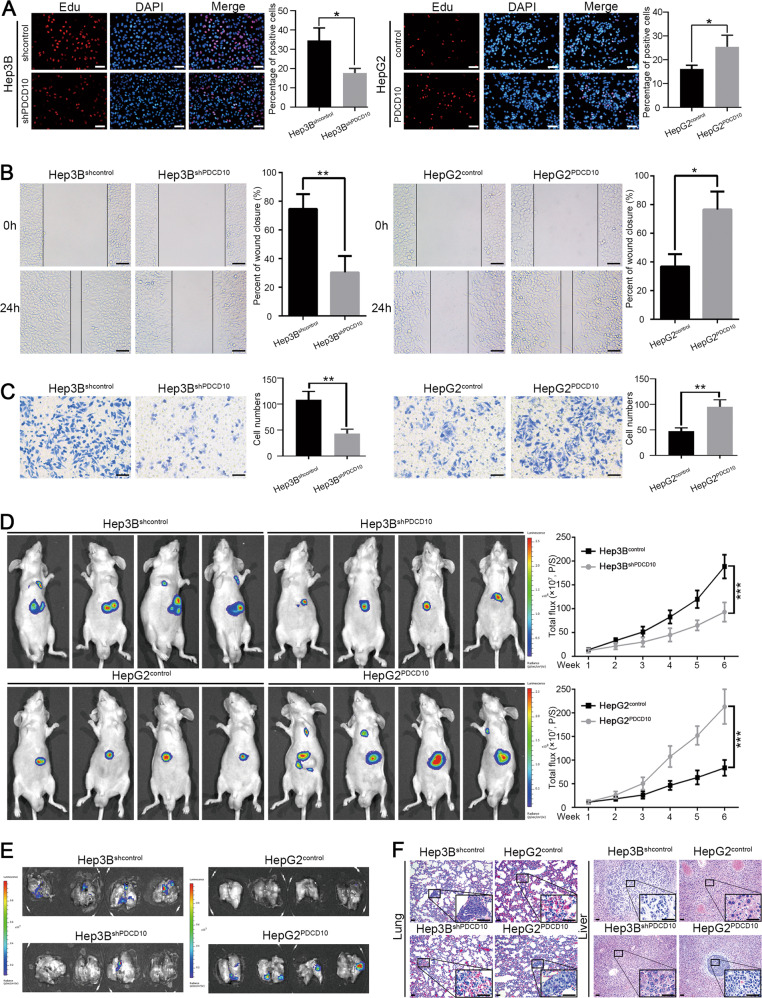
PDCD10 promotes HCC cell migration, and invasion in vitro and growth, and metastasis in vivo. **A** Nuclei of proliferating cells were labelled by EdU (red), and all nuclei of HCC cells were labelled by Hoechst 33342 (blue). The EdU positive rate indicated the proliferation rate of each HCC cells. Scale bars, 50 μm. Data are the mean ± SD of three independent experiments. **B**–**C** Wound healing (**B**) and Transwell (**C**) assays were used to detect migratory and invasive abilities of Hep3B^shPDCD10^, HepG2^PDCD10^ and their control cells, respectively. Scale bars, 50 μm. Data are the mean ± SD of three independent experiments. **D** Mouse orthotopic tumours derived from indicated HCC cells were monitored by in vivo imaging system (IVIS) and the tumour growth curves were depicted in the right column. **E** Ex-vivo bioluminescent images of mouse lungs showed that the pulmonary metastasis of tumour-bearing mice from indicated HCC cells. **F** Representative H&E staining of mouse livers and lungs showed intrahepatic and pulmonary metastatic nodules in each group. Scale bars, 50 μm. *P < 0.05; **P < 0.01; ***P < 0.001.

To ascertain the function of PDCD10 in the malignant phenotypes of HCC, we first knocked down PDCD10 expression in Hep3B cells with relatively high PDCD10 expression and overexpressed PDCD10 in HepG2 cells with low PDCD10 expression. The knockdown and overexpression efficiency were verified by qRT-PCR and western blot (Fig. S2A, B). EdU, MTT, and colony formation assays both showed that knockdown of PDCD10 in Hep3B cells significantly decreased their proliferative capacity, while the proliferation rate of HepG2 cells was obviously increased after overexpressing PDCD10 (Fig. 2A; Fig. S3A, B). Wound healing and Transwell assays showed that Hep3B^shPDCD10^ cells exhibited prominently weaker migratory and invasive abilities than Hep3B^shcontrol^ cells, and HepG2^PDCD10^ cells had a greater ability to migrate and invade than HepG2^control^ cells (Fig. 2B, C). To further explore the role of PDCD10 in vivo, we first established a subcutaneous xenograft tumour model. Knockdown of PDCD10 in Hep3B cells significantly inhibited tumour growth, and PDCD10 overexpression in HepG2 cells promoted the growth of subcutaneous tumours (Fig. S3C). Next, a mouse liver orthotopic xenograft tumour model was constructed, and bioluminescence imaging showed that Hep3B^shPDCD10^ cell-derived orthotopic tumours were significantly smaller and grew more slowly than tumours from Hep3B^shcontrol^ cells, while orthotopic tumours derived from HepG2^PDCD10^ cells were significantly larger and grew faster than tumours from HepG2^control^ cells (Fig. 2D). To better sustain our results, PDCD10 protein expression levels in orthotopic tumours derived from HCC cells with either PDCD10 overexpression or silencing were confirmed by IHC (Fig. S3D). Moreover, ex-vivo imaging of mouse lungs showed that PDCD10 knockdown inhibited lung metastasis of Hep3B cells, while PDCD10 overexpression promoted lung metastasis of HepG2 cells (Fig. 2E). Consistently, serial sections of mouse livers and lungs stained with H&E also revealed more liver and lung metastases in Hep3B^shcontrol^ and HepG2^PDCD10^ cells than in Hep3B^shPDCD10^ and HepG2^control^ cells, respectively (Fig. 2F; Fig. S3E, S3F). Taken together, these data suggested that PDCD10 played an important role in promoting HCC progression in vitro and in vivo.

### PDCD10 facilitates EMT of HCC cells

**Fig. 3:**
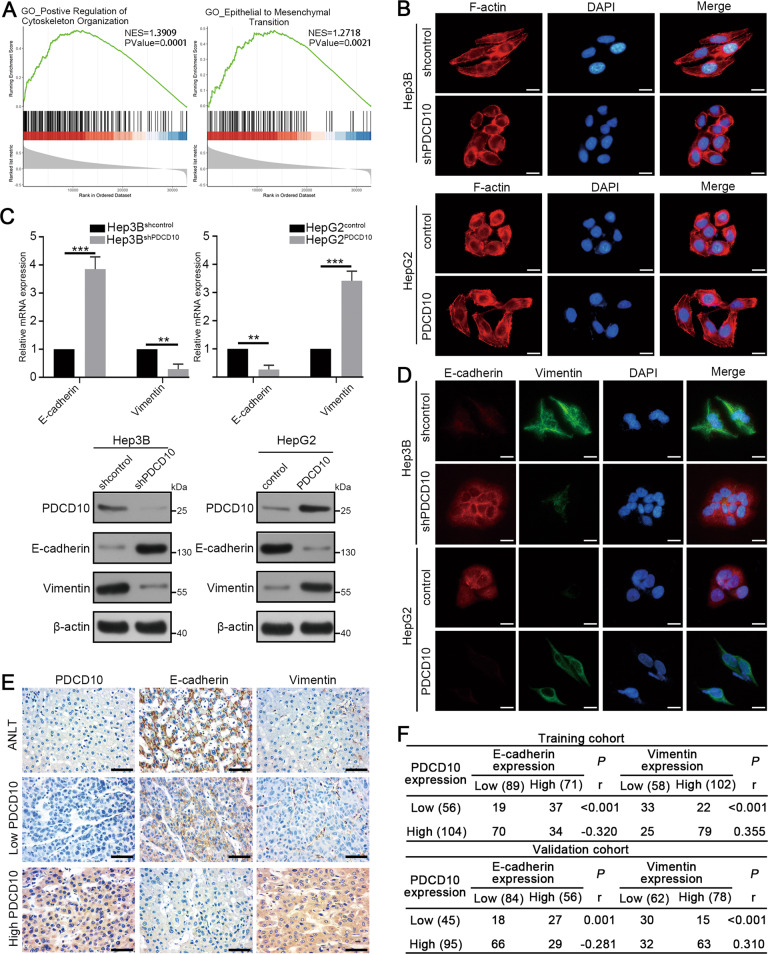
PDCD10 facilitates EMT of HCC cells. **A** GSEA of PDCD10 associated genes in the TCGA HCC database showed that PDCD10 was positively associated with regulation of cytoskeletal organization and EMT. **B** Representative cytoskeletal images of indicated HCC cells. The cytoskeleton was visualized by F-actin staining using rhodamine phalloidin (red). Scale bars, 5 μm. **C** The expression of E-cadherin and vimentin in Hep3B^shPDCD10^, HepG2^PDCD10^, and their control cells was detected by qRT-PCR and western blotting. **D** Immunofluorescence double-staining was used to detect E-cadherin and vimentin simultaneously in indicated HCC cells. Scale bars, 5 μm. (**E**) The representative IHC images showed the expression of E-cadherin and vimentin in ANLT and HCC tissues with high or low PDCD10 expression. Scale bars, 50 μm. **F** The correlation between PDCD10 and E-cadherin, vimentin expression was analysed by Spearman’s rank correlation test in the training and validation cohorts. **P < 0.01; ***P < 0.001.

EMT is one of the key programs in the tumour invasion-metastasis cascade [14] and is usually accompanied by reorganization of the cytoskeleton in tumour cells [15]. Intriguingly, gene set enrichment analysis (GSEA) using TCGA HCC data showed that PDCD10 was positively associated with the regulation of cytoskeletal organization and EMT in HCC (Fig. 3A). Cytoskeletal staining showed Hep3B^shcontrol^ cells with long shuttle forms and elongated F-actin fibres transformed into ovoid forms and contracted F-actin fibres after knocking down PDCD10. However, PDCD10 overexpression led to the opposite transformation in HepG2 cells (Fig. 3B). Then, we further detected EMT markers in HCC cells, and the results showed that the mRNA and protein expression of the epithelial marker E-cadherin was increased and that of the mesenchymal marker vimentin was decreased after knocking down PDCD10 in Hep3B cells, whereas PDCD10 overexpression in HepG2 cells resulted in the opposite results (Fig. 3C). The effects of PDCD10 on EMT markers were further confirmed by using immunofluorescence double-staining of E-cadherin and vimentin (Fig. 3D). Moreover, IHC of HCC serial sections and Spearman’s rank analysis showed that PDCD10 was positively correlated with vimentin, and negatively correlated with E-cadherin in HCC samples in both the training and validation cohorts (Fig. 3E, F). These results collectively demonstrated that PDCD10 influenced the cytoskeleton and promoted EMT in HCC cells.

### PDCD10 promotes YAP activation in HCC

**Fig. 4:**
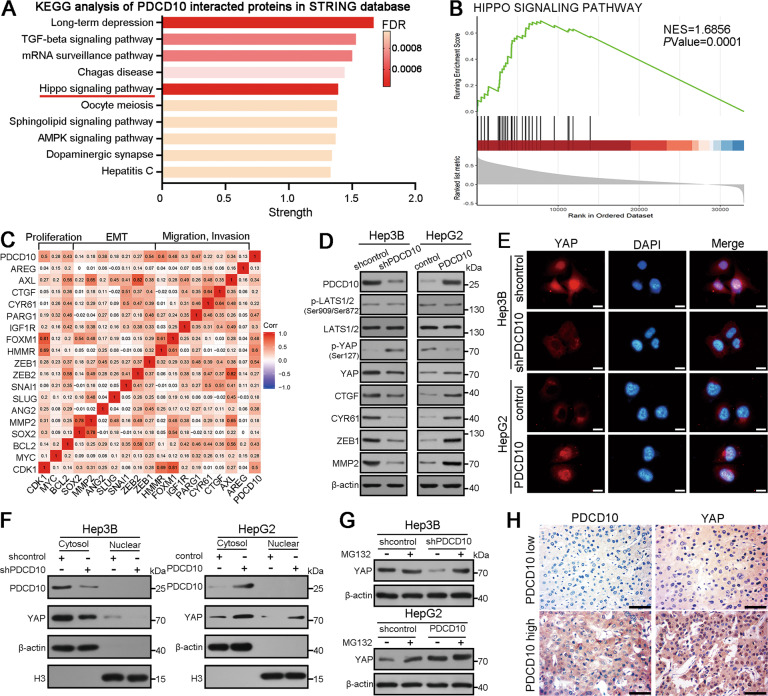
PDCD10 promotes YAP activation in HCC. **A** Kyoto Encyclopedia of Genes and Genomes (KEGG) pathway enrichment analysis of PDCD10 interacted proteins in STRING database (http://string-db.org). **B** GSEA of PDCD10 associated genes in TCGA HCC database showed that PDCD10 was associated with the Hippo signalling pathway. **C** The correlation heatmap (correlation coefficient) using TCGA HCC data showed the correlation between PDCD10 and Hippo signalling downstream effectors associated with tumour proliferation, migration, invasion, and EMT. **D** Western blot analysis of Hippo key proteins and representative downstream effectors in Hep3B^shPDCD10^, HepG2^PDCD10^ and their control cells. **E** Immunofluorescence was used to detect YAP expression in indicated HCC cells. Scale bars, 5 μm. **F** Western blot analysis of PDCD10 and YAP in indicated HCC cells using nuclear–cytoplasmic fractionations. **G** YAP expression was detected in HCC cells by western blotting after treating with proteasomal inhibitor MG132 (10 μM for 6 h). **H** The representative IHC images showed the expression of YAP in HCC tissues with high or low PDCD10 expression. Scale bars, 50 μm.

To gain insight into the molecular mechanism of PDCD10 in promoting HCC progression, we first searched for PDCD10-interacting proteins in the STRING database (http://string-db.org) and performed Kyoto Encyclopaedia of Genes and Genomes (KEGG) pathway enrichment analysis. The full list of PDCD10-interacting proteins was provided in Table S4. Notably, the Hippo signalling pathway attracted our attention, and GSEA using TCGA HCC data revealed that PDCD10 was related to the Hippo signalling pathway (Fig. 4A, B). The Hippo signalling pathway plays a critical role in tumour progression by regulating the expression of many effector molecules involved in tumour proliferation, invasion, and EMT [16, 17]. Correlation analysis using TCGA HCC data showed that PDCD10 was markedly correlated with many Hippo downstream effectors associated with tumour proliferation, migration, invasion, and EMT (Fig. 4C). When Hippo signalling is activated, signalling through MST1/2 and LATS1/2 phosphorylates and inactivates the core functional protein YAP as well as its paralogue TAZ, which leads to inhibition of transcriptional activity by YAP/TAZ cytoplasmic retention and proteasomal degradation [18]. To determine which component of Hippo signalling was modulated, we performed western blotting and the results showed that knockdown of PDCD10 in Hep3B cells caused no altered phosphorylation and expression of LATS1/2 and its upstream kinases but resulted in YAP hyperphosphorylation and downregulation. Consistently, PDCD10 overexpression inhibited YAP phosphorylation and promoted its expression but not its upstream kinases in HepG2 cells (Fig. 4D; Fig. S4A). In addition, the expression of the YAP target genes CTGF, CYR61, ZEB1, and MMP2 was also regulated at both the mRNA and protein levels (Fig. 4D; Fig. S4B). However, the mRNA expression of YAP was consistent after manipulation of PDCD10 expression (Fig. S4B). Although further analysis showed that there was a positive correlation between PDCD10 and YAP mRNA in the Roessler Liver 2, Mas Liver, GSE57957, and TCGA datasets and not in the GSE57957 dataset (Fig. S4C), there was no significant difference in YAP mRNA expression between normal liver and HCC tumour tissues (Fig. S4D) and there was also no significant difference in prognosis for HCC patients stratified by YAP mRNA expression (Fig. S4E). These results indicated that PDCD10 regulates YAP expression at the posttranscriptional level. Nuclear YAP binds TEAD to initiate the transcriptional program. Luciferase reporter assays showed that PDCD10 could promote TEAD transcriptional activity in HCC cells (Fig. S4F). The nuclear translocation of YAP is necessary for YAP-mediated transcription. Intriguingly, PDCD10 knockdown in Hep3B cells caused YAP downregulation and YAP cytoplasmic retention, while PDCD10 overexpression increased the total and nuclear YAP levels in HepG2 cells, as evidenced by immunofluorescence (Fig. 4E) and nuclear–cytoplasmic fractionation (Fig. 4F). Moreover, the proteasome inhibitor MG132 rescued the reduction in YAP levels induced by PDCD10 silencing (Fig. 4G). IHC of HCC tissues from both the training and validation cohorts also showed that there was a positive correlation between PDCD10 expression and YAP nuclear localisation (Fig. 4H; Fig. S4G). In conclusion, these data demonstrated that PDCD10 could promote YAP protein stabilization and nuclear translocation and further expression of downstream target genes.

### PP2Ac is necessary for PDCD10-mediated YAP activation

**Fig. 5:**
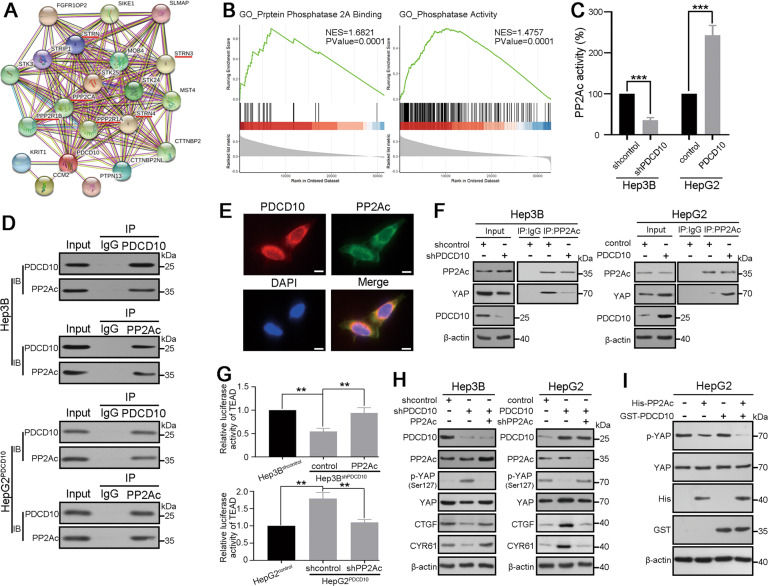
PP2Ac is necessary for PDCD10-mediated YAP activation. **A** The protein-protein interaction network of PDCD10 was constructed in STRING database (http://string-db.org). **B** GSEA of PDCD10 associated genes in TCGA HCC database showed that PDCD10 was associated with protein phosphatase 2A binding and phosphatase activity. **C** The enzymatic activity of PP2Ac in Hep3B^shPDCD10^, HepG2^PDCD10^ and their control cells. Data are the mean ± SD of three independent experiments. **D** Co-immunoprecipitation assay was carried out to determine the direct binding between PDCD10 and PP2Ac in Hep3B and HepG2^PDCD10^ cells. **E** Double immunofluorescence staining showed the colocalization of PDCD10 and PP2Ac in Hep3B cells. Scale bars, 5 μm. **F** Co-immunoprecipitation analysis of PP2Ac and YAP in Hep3B^shPDCD10^, HepG2^PDCD10^ and their control cells. **G** Double luciferase reporter assays showed the TEAD transcriptional activity in PDCD10-interfered HCC cells with PP2Ac knockdown or ectopic expression. Data are the mean ± SD of three independent experiments. **H** Western blot analysis of p-YAP, YAP, CTGF and CYR61 in PDCD10-interfered HCC cells with PP2Ac knockdown or ectopic expression. **I** In vitro dephosphorylation assay was performed to test the effect of PDCD10 on dephosphorylation ability of PP2Ac. **P < 0.01; ***P < 0.001.

To ascertain how PDCD10 promotes YAP activation, we analysed the protein–protein interaction of PDCD10 in the STRING database, and GO functional enrichments showed that PDCD10 was significantly associated with the protein phosphatase type 2A (PP2A) complex and PP2A binding (Fig. 5A; Fig. S5A). GSEA using TCGA HCC data also showed that PDCD10 was associated with PP2A binding and phosphatase activity (Fig. 5B). The PP2A complex is a serine/threonine phosphatase and comprises scaffolding subunit “A” (PP2Aa), regulatory subunit “B”, and catalytic subunit “C” (PP2Ac) [19]. A previous study demonstrated that PP2Ac could interact with YAP and was able to efficiently dephosphorylate and activate YAP [20]. Based on these facts, we speculated that PDCD10 may regulate PP2Ac to activate YAP. Knockdown of PDCD10 in Hep3B cells resulted in decreased activity of PP2Ac, while PDCD10 overexpression increased PP2Ac activity in HepG2 cells (Fig. 5C). Co-IP results revealed that PDCD10 and PP2Ac could interact with each other in HCC cells (Fig. 5D). Immunofluorescence results also showed that PDCD10 colocalized with PP2Ac in the cytoplasm of Hep3B cells (Fig. 5E). Moreover, in Hep3B cells, knockdown of PDCD10 decreased the interaction of PP2Ac and YAP, while PDCD10 overexpression increased their interaction in HepG2 cells (Fig. 5F). Then, we overexpressed PP2Ac in Hep3B^shPDCD10^ cells and transfected HepG2^PDCD10^ cells with PP2Ac shRNA lentivirus (Fig. S5B). Luciferase reporter assays showed that the decreased TEAD transcriptional activity of Hep3B^shPDCD10^ cells was recovered after ectopic expression of PP2Ac, and the enhanced TEAD transcriptional activity of HepG2^PDCD10^ cells was decreased after knockdown of PP2Ac (Fig. 5G). As expected, western blot analysis showed that PP2Ac decreased the p-YAP level and rescued YAP expression in Hep3B^shPDCD10^ cells. The p-YAP level was elevated and total YAP was decreased in HepG2^PDCD10^ cells after knocking down PP2Ac. The downstream effectors CTGF, and CYR61 also showed a corresponding tendency (Fig. 5H). An in vitro dephosphorylation assay revealed that PP2Ac was able to dephosphorylate YAP, which was more obvious in the presence of PDCD10 (Fig. 5I). In summary, PDCD10 interacted with PP2Ac, increased its enzyme activity and further promoted the interaction between PP2Ac and YAP, which led to YAP dephosphorylation and activation.

### PP2Ac is critical for PDCD10-mediated HCC progression

**Fig. 6:**
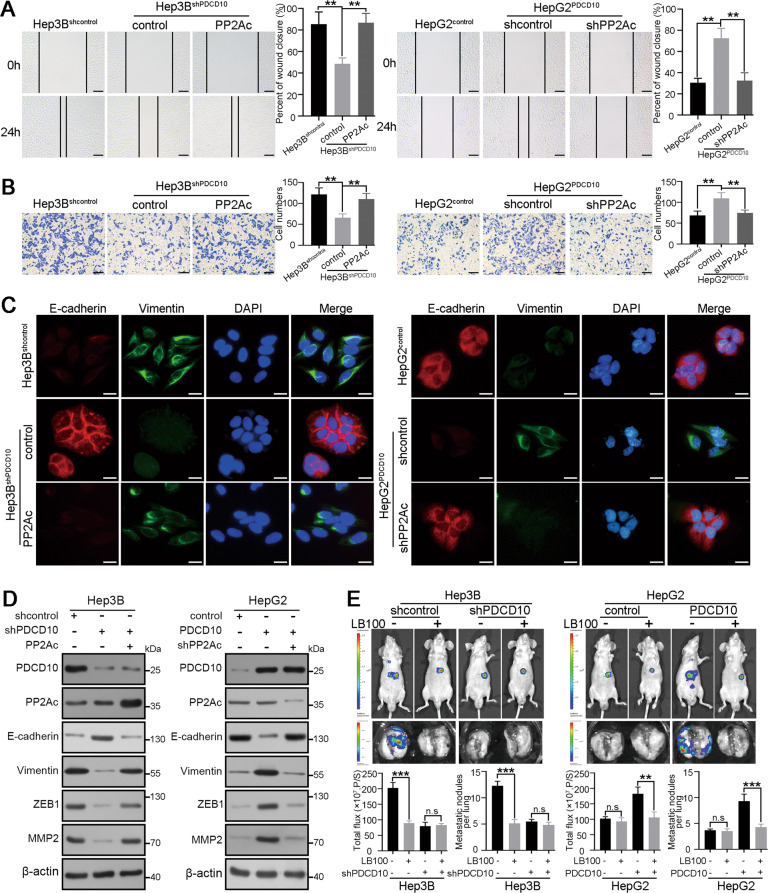
PP2Ac is critical for PDCD10-mediated HCC progression. **A**,**B** Wound healing (**A**) and Transwell assays (**B**) showed the role of PP2Ac in mediating the influence of PDCD10 on HCC cell migratory and invasive capacity. Data are the mean ± SD of three independent experiments. Scale bars, 50 μm. **C** E-cadherin and vimentin expression in PDCD10-interfered HCC cells with PP2Ac further overexpression or knockdown were detected using double immunofluorescence staining. Scale bars, 5 μm. **D** Protein expression of EMT markers in PDCD10-interfered HCC cells with PP2Ac further overexpression or knockdown. **E** Representative bioluminescent images of mouse orthotopic tumours and ex-vivo lungs derived from indicated HCC cells with or without PP2Ac inhibitor LB-100 treatment (2.5 mg/kg every other day from 1 week after the model construction by intraperitoneal injection). Luciferase activity of orthotopic tumours and the number of metastatic nodules in lungs were compared in the lower panel (*n* = 4). n.s, no significance; **P < 0.01; ***P < 0.001.

**Fig. 7:**
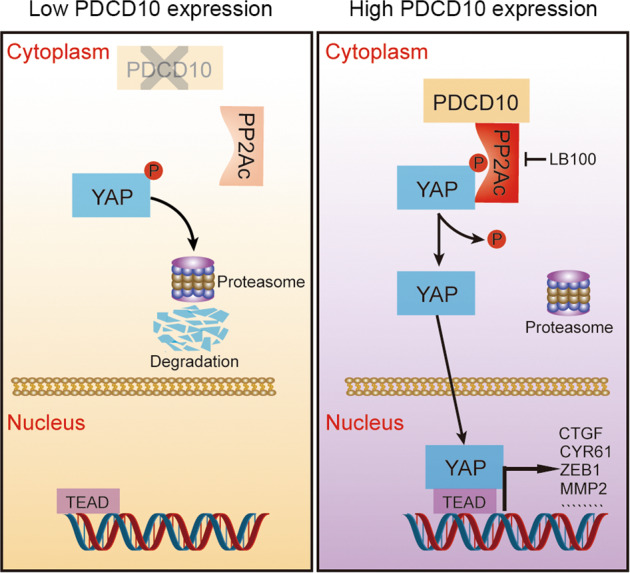
Schematic diagram depicted the mechanism of PDCD10-mediated YAP activation and HCC progression. In HCC with low PDCD10 expression, PP2Ac is devitalized and separated from YAP, leading to YAP hyperphosphorylation and degradation by proteasome. While in HCC with high PDCD10 expression, PDCD10 enhances enzymatic activity of PP2Ac and promotes the interaction between PP2Ac and YAP, leading to YAP dephosphorylation and nuclear translocation, which finally promotes HCC progression by inducing downstream gene expression. LB100 could prevent HCC progression by inhibiting PP2Ac activity.

To further investigate whether PP2Ac is indispensable for PDCD10 induced HCC progression, we first performed in vitro wound healing and Transwell assays. The results showed that PP2Ac overexpression in Hep3B^shPDCD10^ cells restored its decreased migration and invasion capacity, while knockdown of PP2Ac in HepG2^PDCD10^ cells attenuated its high migration and invasion ability (Fig. 6A, B). E-cadherin and vimentin in Hep3B^shPDCD10^ cells recovered to levels in Hep3B^shcontrol^ cells after overexpression of PP2Ac, and EMT markers in HepG2^PDCD10^ cells were restored after knockdown of PP2Ac (Fig. 6C). Furthermore, the role of PP2Ac in PDCD10-mediated EMT was tested by western blot and qRT-PCR (Fig. 6D; Fig. S6A). LB-100 is a novel potent inhibitor of PP2A, and a recently completed phase I study confirmed its antitumour activity in many solid tumours [21]. Considering the high PP2Ac activity in HCC cells with high PDCD10 expression, we asked whether LB-100 could reduce the tumour-promoting function of PDCD10. In vitro assays showed that LB-100 (10 μM) significantly decreased PP2Ac activity in high PDCD10 expressing cells but had no role in PDCD10 deficient HCC cells (Fig. S6B). Then, we further carried out animal experiments to examine whether LB-100 could inhibit HCC progression caused by PDCD10 upregulation. Intriguingly, intraperitoneal injection of LB-100 at 2.5 mg/kg significantly inhibited tumour growth and lung metastasis of orthotopic tumours derived from Hep3B^shcontrol^ and HepG2^PDCD10^ cells but had no significant effects on HCC cells with low PDCD10 expression (Fig. 6E). In short, the role of PDCD10 in promoting HCC migration, EMT, and metastasis was mediated by PP2Ac, and LB-100 may be a potent inhibitor to prevent the progression of HCC with high PDCD10 expression. Therefore, the above data collectively depict a molecular model for PDCD10 in the promotion of HCC cell invasion and metastasis, as illustrated in Fig. 7.

## Discussion

Metastasis-associated recurrence is still the major cause of poor prognosis for HCC patients. Thus, it is of great importance to better understand the underlying molecular mechanisms and develop new targeted therapeutics for HCC patients. Many public datasets and our research findings provide evidence that PDCD10 is frequently upregulated in HCC tissues and associated with poor prognosis in HCC patients. Of note, much higher PDCD10 expression was observed in HCC patients with MVI or metastatic potential. In addition, high PDCD10 expression was associated with poor clinicopathological characteristics and was also an independent risk factor for both OS and DFS. Previous studies revealed that PDCD10 not only regulates apoptosis and proliferation of cells but also plays an important role in migration and motility [22, 23]. Indeed, functional experiments showed that PDCD10 promotes HCC cell proliferation, migration and invasion in vitro and tumour growth and metastasis in vivo, which confirmed the oncogenic role of PDCD10 in HCC metastatic progression.

EMT is considered a hallmark of cancer and drives the transformation of epithelial cells to a mesenchymal-like or a mesenchymal phenotype, leading to the dissemination of tumour cells [24, 25]. There are a few studies that have referred to the potential promoting role of PDCD10 in EMT at present [26, 27]. In this study, we determined cytoskeletal changes, induction of mesenchymal markers, and inhibition of epithelial markers after overexpressing PDCD10 in HCC cells, whereas loss of PDCD10 led to the opposite results. Moreover, a positive correlation between PDCD10 and vimentin and a negative correlation between PDCD10 and E-cadherin were observed in HCC clinical samples. These results provide direct evidence that PDCD10 participates in EMT in HCC. Tan et al. [10] demonstrated that PDCD10 degradation could cause mesenchymal to amoeboid transition (MAT), leading to suppressed collective breast tumour cell migration, namely, breast cancer cells with high PDCD10 expression adopt mesenchymal movement. These results both showed that PDCD10 could promote mesenchymal cell migration of tumour cells. In contrast to amoeboid cell migration, mesenchymal migration is characterised by elongated morphology, cell-ECM adhesion and pericellular proteolysis [28]. Cancer cells can transform their migration modes based on different tumour microenvironments. Whether PDCD10 also participates in amoeboid cell migration of HCC cells is an intriguing subject to be studied in the future.

Hippo signalling is a classical tumour suppressor pathway, and compelling evidence suggests that activation of the core effector protein YAP plays an oncogenic role in liver cancer [29]. The activation of the Hippo signalling involves phosphorylation of a range of proteins, which finally leads to phosphorylated YAP cytoplasmic sequestration and proteasomal degradation [30]. Dephosphorylation of YAP contributes to HCC progression by activating the EMT program [31]. Here, we reported that PDCD10 dephosphorylates YAP and promotes its nuclear translocation, resulting in the transcription of downstream target genes. Given the lack of enzymatic activity of PDCD10, we postulated that PDCD10 may dephosphorylate YAP in an indirect manner. Considering the close connection between PDCD10 and PP2A, we further hypothesised that PDCD10 may link with PP2A to dephosphorylate YAP, as previous studies have shown that the PP2A complex could directly interact and regulate YAP [20, 32]. These hypotheses were validated by the finding that PDCD10 could interact with PP2Ac and increase its enzymatic activity, which further promotes the interaction and dephosphorylation of YAP. A previous study demonstrated that YAP protein expression in HCC was associated with poor OS [33]. Studies have shown that in HCC, the core Hippo constituents are typically not mutated; instead, the increase in YAP levels is due largely to gene amplification and posttranscriptional regulation [34]. Our results also showed that PDCD10 regulates YAP expression at the posttranscriptional level. Even so, how PDCD10 affects the activity of PP2Ac and whether these interactions involve other proteins need to be further explored. We also found that LB-100, a PP2A inhibitor with anticancer effects in many human malignancies [35], and with the enhancement effect of therapeutics for doxorubicin and cisplatin in HCC [36], significantly inhibited PP2Ac activity and HCC tumour growth and metastasis caused by PDCD10 overexpression, which provided a new way to treat HCC.

In conclusion, we found that high PDCD10 expression was useful for predicting the prognosis of HCC patients. We also proved that PDCD10 promotes invasion and metastasis of HCC cells by facilitating EMT. PDCD10 could dephosphorylate and activate YAP by interacting with PP2Ac and increasing its activity. Therefore, our study suggests that PDCD10 acts as a valuable prognostic biomarker and a potential therapeutic target for HCC.

## Materials and methods

### HCC samples and follow-up study

One hundred and sixty specimens were randomly selected from HCC patients who received curative liver resection in the Department of Surgery, Xiangya Hospital of Central South University from January 2007 to December 2012, and further enrolled into the training cohort. One hundred and forty specimens in the validation cohort collected from January 2008 to December 2012 were randomly selected from HCC patients who received liver resection at the Department of Abdominal Surgical Oncology, Affiliated Cancer Hospital of Xiangya School of Medicine, Central South University. The details for the patient enrolment were shown in Fig. S1A. In addition, another 30 pairs of randomly selected snap-frozen HCC tissues and adjacent non-tumorous liver tissues (ANLTs) were obtained between January 2011 and December 2013 at the Department of Surgery, Xiangya Hospital, and used to quantitative real-time PCR (qRT-PCR) and western blot analysis. Follow-up procedures were conducted as described in our previous study [37]. OS was defined as the interval between the date of liver resection and death of HCC or the last follow-up. DFS was defined as the time interval between the date of surgery and recurrence or metastasis. The diagnosis of patients was confirmed by two independent pathologists. All researches followed the REMARK guidelines for reporting prognostic biomarkers in cancer [38]. This study was approved by the ethics committee of Xiangya Hospital and Affiliated Cancer Hospital of Xiangya School of Medicine at Central South University. Informed consent in writing was obtained from each patient and the study protocol conformed to the ethical guidelines of the 1975 Declaration of Helsinki.

### Cell lines and cell culture

MHCC97-L, MHCC97-H and HCCLM3 were kindly provided by the Liver Cancer Institute of Fudan University, Shanghai, China. PLC/PRF/5, Hep3B and HepG2 cells were purchased from the American Type Culture Collection (ATCC, Manassas, VA). L02, SMMC7721 and Huh7 cells were purchased from the Cell Bank of Typical Culture Preservation Committee of Chinese Academy of Science, Shanghai, China. Short tandem repeat (STR) DNA fingerprinting was used to authenticate all cell lines before experiments. The cell lines have been regularly tested for mycoplasma contamination using the Universal Mycoplasma Detection Kit obtained from ATCC (Manassas, VA). Cell lines were cultured in high glucose Dulbecco’s modified eagle media (BioInd, Beit HaEmek, Israel) supplemented with 10% fetal bovine serum (FBS, BioInd) and 1% penicillin-streptomycin (Hyclone, Logan, UT) and maintained in a 37 °C incubator with 5% CO_2_. For MG132 treatment, the cells were cultured in the presence or absence of 10 μM MG132 (Beyotime Biotechnology, Shanghai, China) for 6 h.

### RNA extraction and quantitative real-time PCR (qRT-PCR)

Total RNA was extracted from tissues or cells with TRIzol reagent (Invitrogen, Carlsbad, CA) according to the manufacturer’s protocol. Reverse transcription was performed using the universal cDNA synthesis kit (Exiqon, Vedbaek, Denmark). qRT-PCR was performed on PRISM 7300 Sequence Detection System (Applied Biosystems, CA) using FastStart Universal SYBR Green Master Kit (Roche Life Sciences, Switzerland). GAPDH was used as an internal control. The relative expression level was quantified by 2^−ΔCt^ or 2^−ΔΔCt^ method. All experiments were done in triplicates. The primer sequences were listed in the Table S5.

### Protein extraction and western blot

Total proteins were extracted with RIPA lysis buffer adding 1% protease inhibitor cocktail (CWBIO, Beijing, China). For cytoplasmic and nuclear proteins extraction, the Nuclear and Cytoplasmic Extraction Kit (CWBIO, Beijing, China) was used according to the manufacturer’s instruction. Identical quantities of proteins were then separated by sodium dodecyl sulfate-polyacrylamide gel electrophoresis (SDS-PAGE) and further transferred onto PVDF membrane (Millipore, Bedford, MA). The membrane was blocked with 5% skimmed milk and incubated with the appropriate antibody. The antigen–antibody complex on the membrane was detected by enhanced chemiluminescence reagents (Thermo Scientific, Waltham, MA). The experiments were done in triplicates. All the primary antibodies and secondary antibodies were listed in Tables S6 and S7.

### Immunohistochemistry (IHC)

Formalin-fixed paraffin sections of HCC were stained using the universal two-step detection kit (ZSGB-BIO, Beijing, China). IHC experiments were conducted as previously described [37]. The IHC score of target proteins was evaluated according to the proportion and intensity of positive cells [39, 40]. In brief, staining intensity was classified as: 0, negative; 1, weak; 2, moderate; 3, strong. Percentage of positive cells was defined as: 0, < 5% positive cells; 1, 5 ~ 25% positive cells; 2, 26 ~ 50% positive cells; 3, 51 ~ 75% positive cells; 4, > 75% positive cells. Therefore, the value range of the immunostaining score was 0 ~ 12 points. The high protein expression was defined as score ≥ 4, while low protein expression was defined as score < 4. The antibodies were listed in the Table S6. The slides were independently evaluated by two researchers.

### Vector construction and transfection

The ectopic expression and knockdown lentivirus as well as control lentivirus were purchased from Vigene Biosciences (Shandong, China). For overexpression studies, full-length cDNA of PDCD10 and PP2Ac were inserted into lentiviral vectors. Lentivirus-containing short hairpin RNAs (shRNAs) targeting PDCD10 or PP2Ac were transfected into corresponding HCC cells according to the manufacturer’s instructions. Cells transfected with empty vectors were used as controls. Puromycin (Sigma, St. Louis, MO; 3 μg/mL) was used to select stable clones. Overexpression or depletion of the target gene was verified by qRT-PCR and western blot analysis. The shRNA with the most significant knockdown efficacy was selected for subsequent experiments. The sequences of the shRNA were listed in Table S8. All transfections were performed using Lipofectamine 2000 (Invitrogen, Carlsbad, CA).

### MTT assay and colony formation assay

For MTT assay, an equal number of HCC cells was seeded into 96-well plates and incubated for successive 7 days. The cells were stained with MTT (Sigma, St Louis, MO) and subjected to determine absorbance values at 570 nm using a spectrophotometer. For colony formation assay, 500 HCC cells were added into each well of 6-well plates and incubated in 5% CO_2_ at 37 °C for 2 weeks. Then, the colonies were fixed with methanol and stained with 0.1% crystal violet (Beyotime Biotechnology, Shanghai, China). All studies were conducted with three replicates.

### EdU assay

The EdU assay was conducted using Cell-Light EdU Apollo567 In Vitro Kit (RiboBio, Guangzhou, China). 1 × 10^5^ HCC cells were seeded into a 96-well plate and cultured with 100 μl medium containing 50 μM EdU for 2 h. Cells were then fixed and stained according to the manufacturer’s protocol. The results were captured under an inverted fluorescence microscope and the percentage of EdU positive cells was calculated and compared. All experiments were done in triplicates.

### Wound healing assay and Transwell invasion assay

For wound healing assay, HCC cells were seeded into six-well plates and cultured in DMEM medium. Mitomycin-C (10 μg/ml, Sigma, St. Louis, MO) was used to suppress cell proliferation before scratching. The wound was made through the monolayer cell with a 10 μl sterile pipette tip and cells were cultured with the replaced medium. The percent of wound closure was calculated after 24 h. For Transwell invasion assay, cells were preincubated with mitomycin-C (10 μg/ml, Sigma, St. Louis, MO) for 1 h at 37 °C to suppress proliferation and about 1 × 10^5^ cells in 200 μL of serum-free medium were seeded into the upper chamber of the Transwell insert with matrigel (BD Biosciences, Franklin Lakes, NJ). After 24 h of incubation, cells that invaded to the bottom membrane were fixed with methanol and stained with 0.1% crystal violet (Beyotime Biotechnology, Shanghai, China). The scratch and the numbers of invaded cells were captured under an inverted microscope. Three random fields were chosen for counting and all assays were performed in triplicates.

### Bioinformatic data mining

The gene expression data of PDCD10 and YAP in TCGA HCC tissues were downloaded from the UCSC Xena browser (http://xena.ucsc.edu/) [41]. We also analysed PDCD10 mRNA expression in HCC tissues and control samples using the GSE57957 [42] and GSE36376 [43] datasets in the GEO. Survival data from TCGA HCC data in the UCSC Xena browser were used to analyse the influence of PDCD10 and YAP on prognosis of HCC patients. The gene expression profiles in the TCGA HCC dataset were subject to GSEA using the clusterProfiler R package [44], false discovery rate (FDR) < 0.25 and *P* < 0.05 were set as the cut-off criteria. The protein–protein interaction (PPI) network was constructed and analysed using STRING online database (version 11; http://string-db.org) [45] with high interaction score (0.700) and no more than 20 interactors to show.

### Immunofluorescence

About 5 × 10^5^ cells seeded into the six-well culture plate were grown at glass coverslip. The coverslip was fixed with 4% paraformaldehyde and then incubated with 0.5% Triton X-100. After incubation with primary antibody overnight at 4 °C, appropriate fluorescence labelled secondary antibodies were used to detect corresponding proteins. The primary and secondary antibodies were listed in Tables S5 and S6. For cytoskeleton analysis, F-actin was labelled with rhodamine-conjugated phalloidin (Roche, Basel, Switzerland) according to the manufacturer’s protocol. DAPI (Beyotime, Shanghai, China) was used to stain nuclei. All experiments were done in triplicates.

### HCC mouse model and in vivo imaging study

The HCC model in mouse was constructed as previously described [37, 46]. Animal xenograft experiments were conducted with 4-week-old male BALB/c nude mice. Before the experiment, HCC cells were transfected with vectors that express luciferase under the help of Science Light Biotechnology (Shanghai, China). For the subcutaneous tumour model, around 5 × 10^6^ cells within 0.2 mL cool PBS were injected subcutaneously into the left upper flank region of nude mice for 4 weeks (4 in each group). For the orthotopic tumour model, the subcutaneous tumours were resected and cut into pieces of approximately 1 mm^3^ and implanted into the liver of nude mice (4 in each group). The tumour formation and metastasis were monitored by bioluminescence imaging using the Xenogen IVIS imaging system 100 (Caliper Life Sciences, Hopkinton, MA). In brief, mice were injected intraperitoneally with D-Luciferin potassium salt (Sciencelight Biotechnology, Shanghai, China) at 100 mg/kg and then subjected to imaging. The mice were sacrificed 6 weeks later, and the livers and lungs were harvested, imaged, and processed for histopathological examination. For in vivo intervention using LB-100, LB-100 (Selleck, Shanghai, China) were injected i.p. for mice born with orthotopic tumours at 2.5 mg/kg every other day from 1 week after the model construction. All mice were selected and allocated randomly. No blinding was performed in this study. All animal experiments were carried out according to the protocols approved by the Medical Experimental Animal Care Commission of CSU and were approved by the Animal Ethics Committee of the Central South University.

### Luciferase reporter assay

For the luciferase reporter assay, HCC cells were seeded on a 24-well culture plate and grown overnight to 70–80% confluency. Then, the cells were transfected with TEAD luciferase reporter plasmid 8xGTIIC-luciferase (Addgene, Catalogue #34615) as well as with pRL-CMV Renilla luciferase plasmid (GeneChem, Shanghai, China) using Lipofectamine LTX (Invitrogen, Carlsbad, CA), according to the manufacturer’s protocol. Twenty-four hours later, cells were harvested and lysed and analysed for luciferase activities using a Dual-luciferase Reporter Assay System (Promega, Madison, WI) according to the manufacturer’s protocol. The activity of firefly luciferase was normalised by that of the Renilla luciferase activity. All experiments were done in triplicates.

### PP2A phosphatase activity assay

PP2A activity was determined using PP2A immunoprecipitation phosphatase assay kit (Millipore, Darmstadt, Germany) according to the manufacturer’s instructions. Briefly, cells were lysed and the total protein was immunoprecipitated with anti-PP2A, C subunit antibody along with Protein A Agarose. The activity of PP2Ac was assessed by dephosphorylation of the phosphopeptide (K-R-pT-I-R-R). The phosphates released from the dephosphorylation of phosphopeptide were detected by malachite green and the relative absorbance was measured at 640 nm in a microtiter plate reader. All experiments were done in triplicates.

### Co-immunoprecipitation (co-IP)

Co-IP was performed using a Pierce Co-IP kit (Thermo Fisher Scientific, Waltham, MA) following the manufacturer’s protocol. In brief, monolayer culture cells were first lysed and protein concentration was determined. Then, about 1000ug proteins were incubated with 10 μg of IP antibody or control IgG at 4 °C overnight to form the immune complex. The antigen sample/antibody mixture were mixed with pre-washed magnetic beads at room temperature for 1 h. Finally, the IP targets were disassociated from the beads and samples were analysed by western blotting.

### In vitro dephosphorylation assay

The whole-cell lysate from HepG2 was incubated with or without PP2Ac and PDCD10 fusion protein (Proteintech, Rosemont, IL) at 37 °C for 10 min. The reaction was then stopped by heating at 100 °C for 5 min and loaded on SDS-PAGE to detect p-YAP.

### Statistical analysis

Statistical analyses were performed using SPSS 20.0 and Graphpad Prism 8. Data were expressed as mean ± SD from at least three independent experiments and analysed using Student’s *t*-test or one-way ANOVA. Correlation analysis was determined by Spearman’s rank analysis or Pearson correlation analysis. The Chi-squared test was applied to examine the association between PDCD10 expression and clinicopathologic parameters. OS and DFS curves were depicted by the Kaplan–Meier method and analysed by the log-rank test. Univariate and multivariate analyses with Cox proportional hazards model were used to verify the independent risk factors. The variance is similar between the groups that are being statistically compared. A two-tailed *P*-values less than 0.05 were considered to be of statistical significance.

## Supplementary information

Supplementary Figures

Supplementary Tables

## Data Availability

All the data generated or analysed during this study are included in this published article and its supplementary files.

## References

[1] Bray F, Ferlay J, Soerjomataram I, Siegel RL, Torre LA, Jemal A (2018). Global cancer statistics 2018: GLOBOCAN estimates of incidence and mortality worldwide for 36 cancers in 185 countries. CA Cancer J Clin.

[2] Villanueva A (2019). Hepatocellular Carcinoma. N. Engl J Med.

[3] Forner A, Reig M, Bruix J (2018). Hepatocellular carcinoma. Lancet.

[4] Wang Y, Liu H, Zhang Y, Ma D (1999). cDNA cloning and expression of an apoptosis-related gene, humanTFAR15 gene. Sci China C Life Sci.

[5] Shi Z, Jiao S, Zhou Z (2016). STRIPAK complexes in cell signaling and cancer. Oncogene.

[6] Fidalgo M, Guerrero A, Fraile M, Iglesias C, Pombo CM, Zalvide J (2012). Adaptor protein cerebral cavernous malformation 3 (CCM3) mediates phosphorylation of the cytoskeletal proteins ezrin/radixin/moesin by mammalian Ste20-4 to protect cells from oxidative stress. J Biol Chem.

[7] Zhu Y, Wu Q, Xu JF, Miller D, Sandalcioglu IE, Zhang JM (2010). Differential angiogenesis function of CCM2 and CCM3 in cerebral cavernous malformations. Neurosurg Focus.

[8] Wan X, Saban DV, Kim SN, Weng Y, Dammann P, Keyvani K (2020). PDCD10-deficiency promotes malignant behaviors and tumor growth via triggering EphB4 kinase activity in glioblastoma. Front Oncol.

[9] Zhu Y, Zhao K, Prinz A, Keyvani K, Lambertz N, Kreitschmann-Andermahr I (2016). Loss of endothelial programmed cell death 10 activates glioblastoma cells and promotes tumor growth. Neuro Oncol.

[10] Tan P, Ye Y, He L, Xie J, Jing J, Ma G (2018). TRIM59 promotes breast cancer motility by suppressing p62-selective autophagic degradation of PDCD10. PloS Biol.

[11] De Marco C, Zoppoli P, Rinaldo N, Morganella S, Morello M, Zuccala V (2021). Genome-wide analysis of copy number alterations led to the characterisation of PDCD10 as oncogene in ovarian cancer. Transl Oncol.

[12] Yang D, Wang JJ, Li JS, Xu QY (2018). miR-103 functions as a tumor suppressor by directly targeting programmed cell Death 10 in NSCLC. Oncol Res.

[13] Yang LY, Fang F, Ou DP, Wu W, Zeng ZJ, Wu F (2009). Solitary large hepatocellular carcinoma: a specific subtype of hepatocellular carcinoma with good outcome after hepatic resection. Ann Surg.

[14] Yeung KT, Yang J (2017). Epithelial-mesenchymal transition in tumor metastasis. Mol Oncol.

[15] Yilmaz M, Christofori GEMT (2009). The cytoskeleton, and cancer cell invasion. Cancer Metastasis Rev.

[16] Warren J, Xiao Y, Lamar JM. YAP/TAZ activation as a target for treating metastatic cancer. Cancers (Basel). 2018. 10.3390/cancers10040115*.* 10.3390/cancers10040115 PMC5923370 29642615

[17] Janse VRH, Yang X (2016). The roles of the Hippo pathway in cancer metastasis. Cell Signal.

[18] Moroishi T, Hansen CG, Guan KL (2015). The emerging roles of YAP and TAZ in cancer. Nat Rev Cancer.

[19] Wlodarchak N, Xing Y (2016). PP2A as a master regulator of the cell cycle. Crit Rev Biochem Mol Biol.

[20] Schlegelmilch K, Mohseni M, Kirak O, Pruszak J, Rodriguez JR, Zhou D (2011). Yap1 acts downstream of alpha-catenin to control epidermal proliferation. Cell.

[21] Chung V, Mansfield AS, Braiteh F, Richards D, Durivage H, Ungerleider RS (2017). Safety, tolerability, and preliminary activity of LB-100, an inhibitor of protein phosphatase 2A, in patients with relapsed solid tumors: an open-label, dose escalation, first-in-human, phase I trial. Clin Cancer Res.

[22] Ma X, Zhao H, Shan J, Long F, Chen Y, Chen Y (2007). PDCD10 interacts with Ste20-related kinase MST4 to promote cell growth and transformation via modulation of the ERK pathway. Mol Biol Cell.

[23] Mardakheh FK, Self A, Marshall CJ (2016). RHO binding to FAM65A regulates Golgi reorientation during cell migration. J Cell Sci.

[24] Hanahan D, Weinberg RA (2011). Hallmarks of cancer: the next generation. Cell.

[25] Giannelli G, Koudelkova P, Dituri F, Mikulits W (2016). Role of epithelial to mesenchymal transition in hepatocellular carcinoma. J Hepatol.

[26] Fan L, Lei H, Zhang S, Peng Y, Fu C, Shu G (2020). Non-canonical signaling pathway of SNAI2 induces EMT in ovarian cancer cells by suppressing miR-222-3p transcription and upregulating PDCD10. Theranostics.

[27] Dong C, Fan B, Ren Z, Liu B, Wang Y. CircSMARCA5 Facilitates the progression of prostate cancer through miR-432/PDCD10 axis. Cancer Biother Radiopharm. 2020. 10.1089/cbr.2019.3490. 10.1089/cbr.2019.3490 32407167

[28] Wu JS, Jiang J, Chen BJ, Wang K, Tang YL, Liang XH (2021). Plasticity of cancer cell invasion: patterns and mechanisms. Transl Oncol.

[29] Yimlamai D, Fowl BH, Camargo FD (2015). Emerging evidence on the role of the Hippo/YAP pathway in liver physiology and cancer. J Hepatol.

[30] Moon S, Yeon PS, Woo PH (2018). Regulation of the Hippo pathway in cancer biology. Cell Mol Life Sci.

[31] Huang Z, Zhou JK, Wang K, Chen H, Qin S, Liu J (2020). PDLIM1 inhibits tumor metastasis through activating Hippo signaling in hepatocellular carcinoma. Hepatology.

[32] Hein AL, Brandquist ND, Ouellette CY, Seshacharyulu P, Enke CA, Ouellette MM, et al. PR55alpha regulatory subunit of PP2A inhibits the MOB1/LATS cascade and activates YAP in pancreatic cancer cells. Oncogenesis. 2019. 10.1038/s41389-019-0172-9. 10.1038/s41389-019-0172-9 PMC6817822 31659153

[33] Xu MZ, Yao TJ, Lee NP, Ng IO, Chan YT, Zender L (2009). Yes-associated protein is an independent prognostic marker in hepatocellular carcinoma. Cancer-Am Cancer Soc.

[34] Patel SH, Camargo FD, Yimlamai D (2017). Hippo signaling in the liver regulates organ size, cell fate, and carcinogenesis. Gastroenterology.

[35] Hong CS, Ho W, Zhang C, Yang C, Elder JB, Zhuang Z (2015). LB100, a small molecule inhibitor of PP2A with potent chemo- and radio-sensitizing potential. Cancer Biol Ther.

[36] Bai XL, Zhang Q, Ye LY, Hu QD, Fu QH, Zhi X (2014). Inhibition of protein phosphatase 2A enhances cytotoxicity and accessibility of chemotherapeutic drugs to hepatocellular carcinomas. Mol Cancer Ther.

[37] Xiao S, Chang RM, Yang MY, Lei X, Liu X, Gao WB (2016). Actin-like 6A predicts poor prognosis of hepatocellular carcinoma and promotes metastasis and epithelial-mesenchymal transition. Hepatology.

[38] Altman DG, McShane LM, Sauerbrei W, Taube SE (2012). Reporting recommendations for tumor marker prognostic studies (REMARK): explanation and elaboration. PloS Med.

[39] Su S, Liu Q, Chen J, Chen J, Chen F, He C (2014). A positive feedback loop between mesenchymal-like cancer cells and macrophages is essential to breast cancer metastasis. Cancer Cell.

[40] Chui X, Egami H, Yamashita J, Kurizaki T, Ohmachi H, Yamamoto S (1996). Immunohistochemical expression of the c-kit proto-oncogene product in human malignant and non-malignant breast tissues. Br J Cancer.

[41] Goldman MJ, Craft B, Hastie M, Repecka K, McDade F, Kamath A (2020). Visualizing and interpreting cancer genomics data via the Xena platform. Nat Biotechnol.

[42] Mah WC, Thurnherr T, Chow PK, Chung AY, Ooi LL, Toh HC (2014). Methylation profiles reveal distinct subgroup of hepatocellular carcinoma patients with poor prognosis. PloS One.

[43] Lim HY, Sohn I, Deng S, Lee J, Jung SH, Mao M (2013). Prediction of disease-free survival in hepatocellular carcinoma by gene expression profiling. Ann Surg Oncol.

[44] Yu G, Wang LG, Han Y, He QY (2012). clusterProfiler: an R package for comparing biological themes among gene clusters. Omics.

[45] Szklarczyk D, Gable AL, Lyon D, Junge A, Wyder S, Huerta-Cepas J (2019). STRING v11: protein-protein association networks with increased coverage, supporting functional discovery in genome-wide experimental datasets. Nucleic Acids Res.

[46] Chang RM, Xiao S, Lei X, Yang H, Fang F, Yang LY (2017). miRNA-487a promotes proliferation and metastasis in hepatocellular carcinoma. Clin Cancer Res.

