# Supplementary material for: Programmed cell death 10 promotes metastasis and epithelial-mesenchymal transition of hepatocellular carcinoma via PP2Ac-mediated YAP activation
Source: Cell Death Dis. 2021 Sep 14;12(9):849. doi: 10.1038/s41419-021-04139-z (PMC8440642; doi:10.1038/s41419-021-04139-z)
Supplement: Supplementary file 1 — Supplementary Figures [file 41419_2021_4139_MOESM1_ESM.docx]

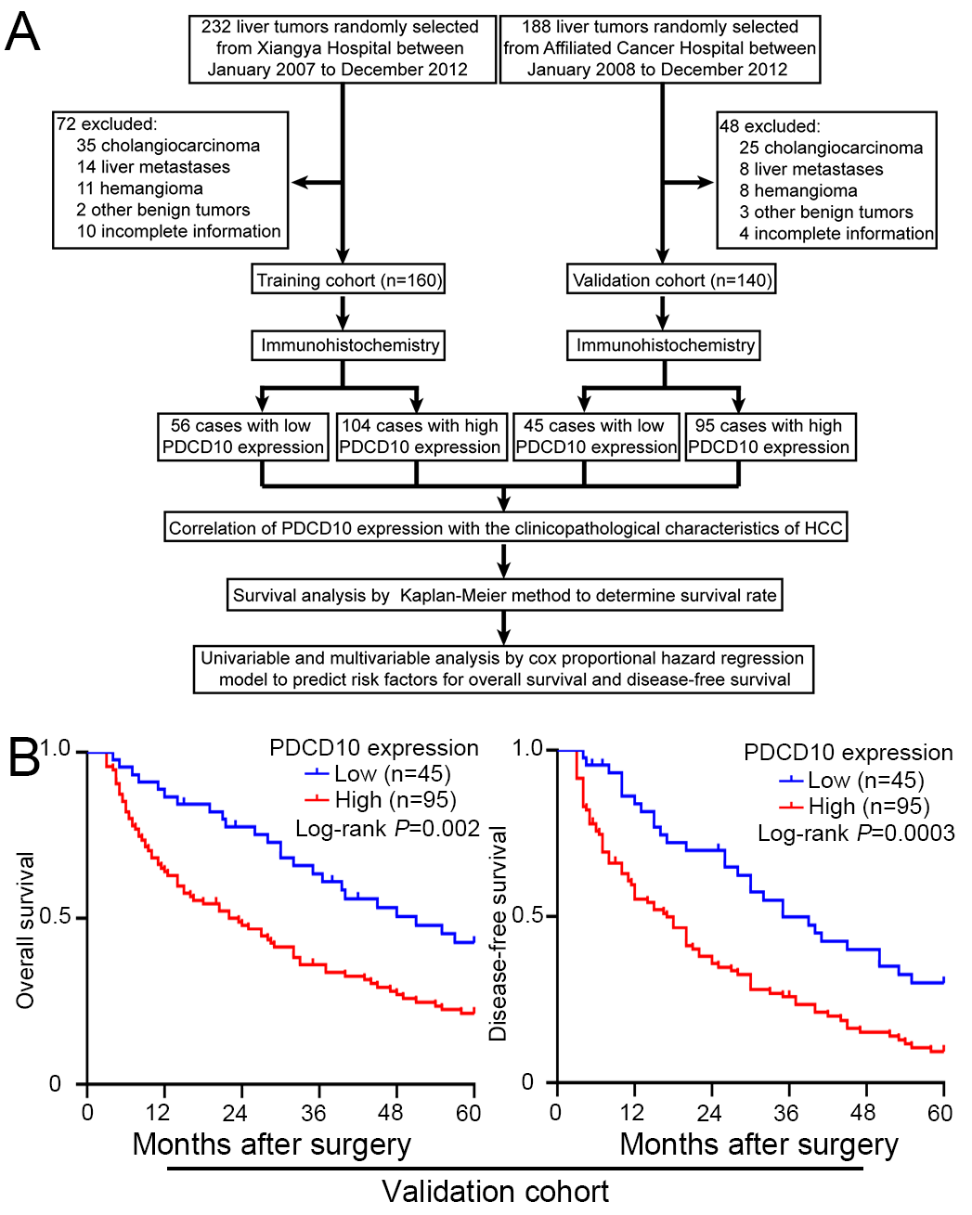


**Fig. S1 PDCD10 is associated with poor prognosis of HCC patients.** (A) Flow chart for clinical study design with two independent cohorts of HCC patients enrolled. (B) Kaplan-Meier analysis of overall survival and disease-free survival of HCC patients with high or low PDCD10 expression in validation cohort.


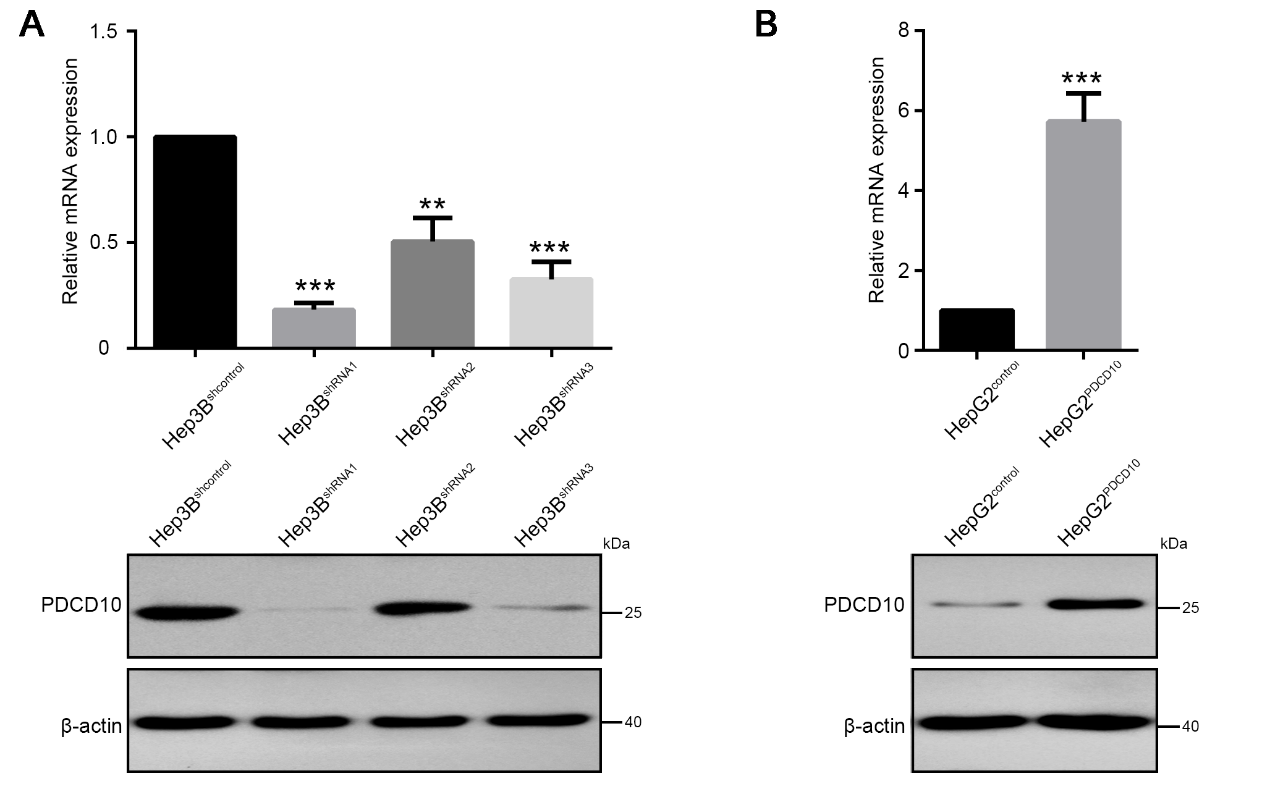


**Fig. S2** **Knockdown and overexpression efficacy were detected by qRT-PCR and western blot.** (A) mRNA and protein expression of PDCD10 in Hep3B cells transfected with lentivirus containing shRNA sequences of PDCD10 or empty vector. (B) mRNA and protein expression of PDCD10 in HepG2 cells transfected with control lentivirus or lentivirus containing ORF sequence of PDCD10. Data are the mean ± SD of three independent experiments. **, *P*<0.01; ***, *P*<0.001.


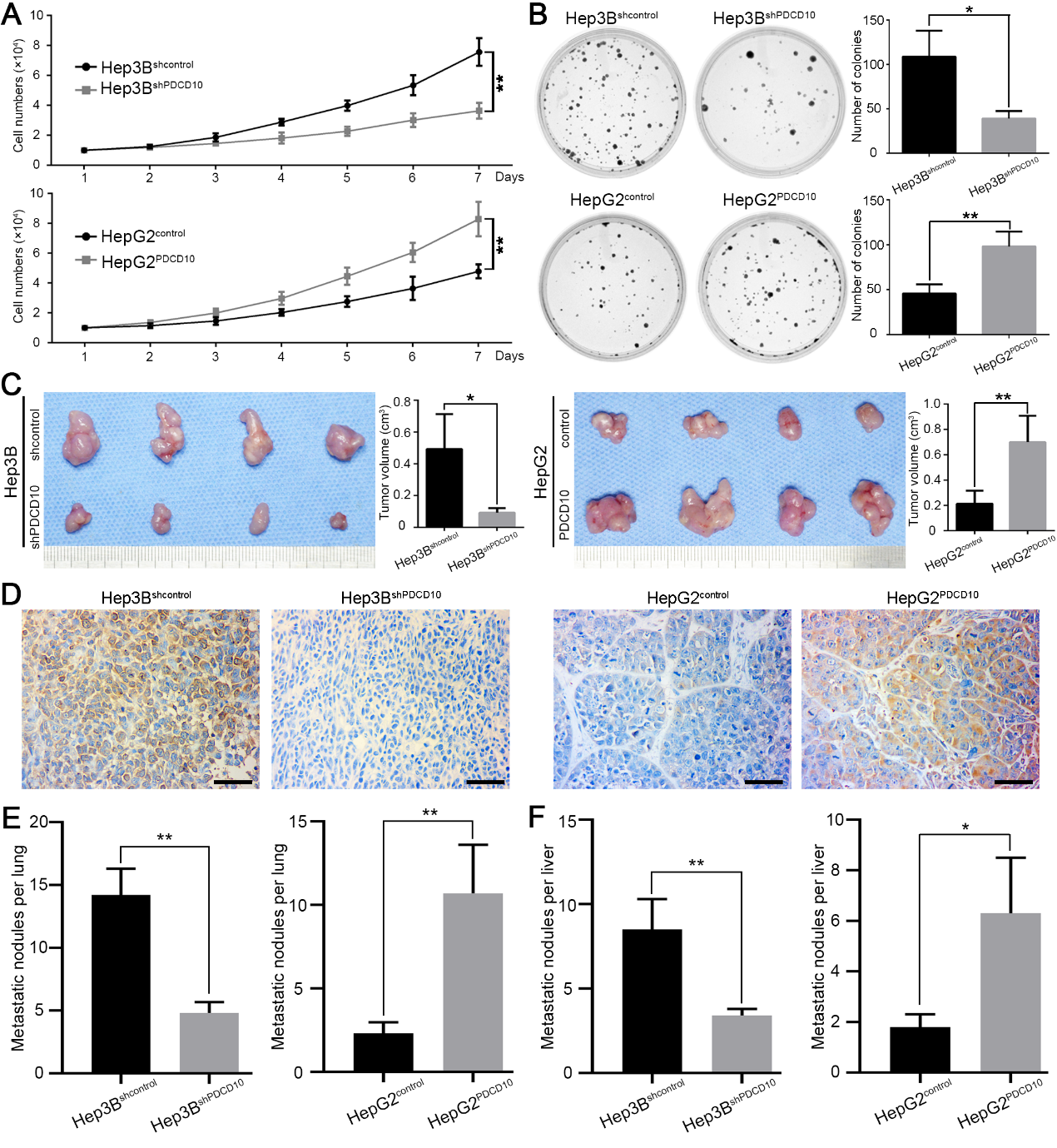


**Fig. S3 PDCD10 promotes HCC cells proliferation, growth and metastasis *in vitro* and *in vivo.*** (A-B) Proliferation ability of Hep3B^shPDCD10^, HepG2^PDCD10^ and their control cells was examined by MTT assay (A) and colony formation assay (B). Data are the mean ± SD of three independent experiments. (C) Subcutaneous tumors derived from Hep3B^shPDCD10^, HepG2^PDCD10^ and their control cells were shown and compared between each group. (D) The expression levels of PDCD10 in orthotopic tumors derived from indicated HCC cells was confirmed by immunohistochemistry. (E) The number of metastatic nodules per lung was calculated and compared between Hep3B^shPDCD10^, HepG2^PDCD10^ and their control cells derived orthotopic tumors. (E) The number of metastatic nodules per liver was calculated and compared between Hep3B^shPDCD10^, HepG2^PDCD10^ and their control cells derived orthotopic tumors. *, *P*<0.05; **, *P*<0.01.


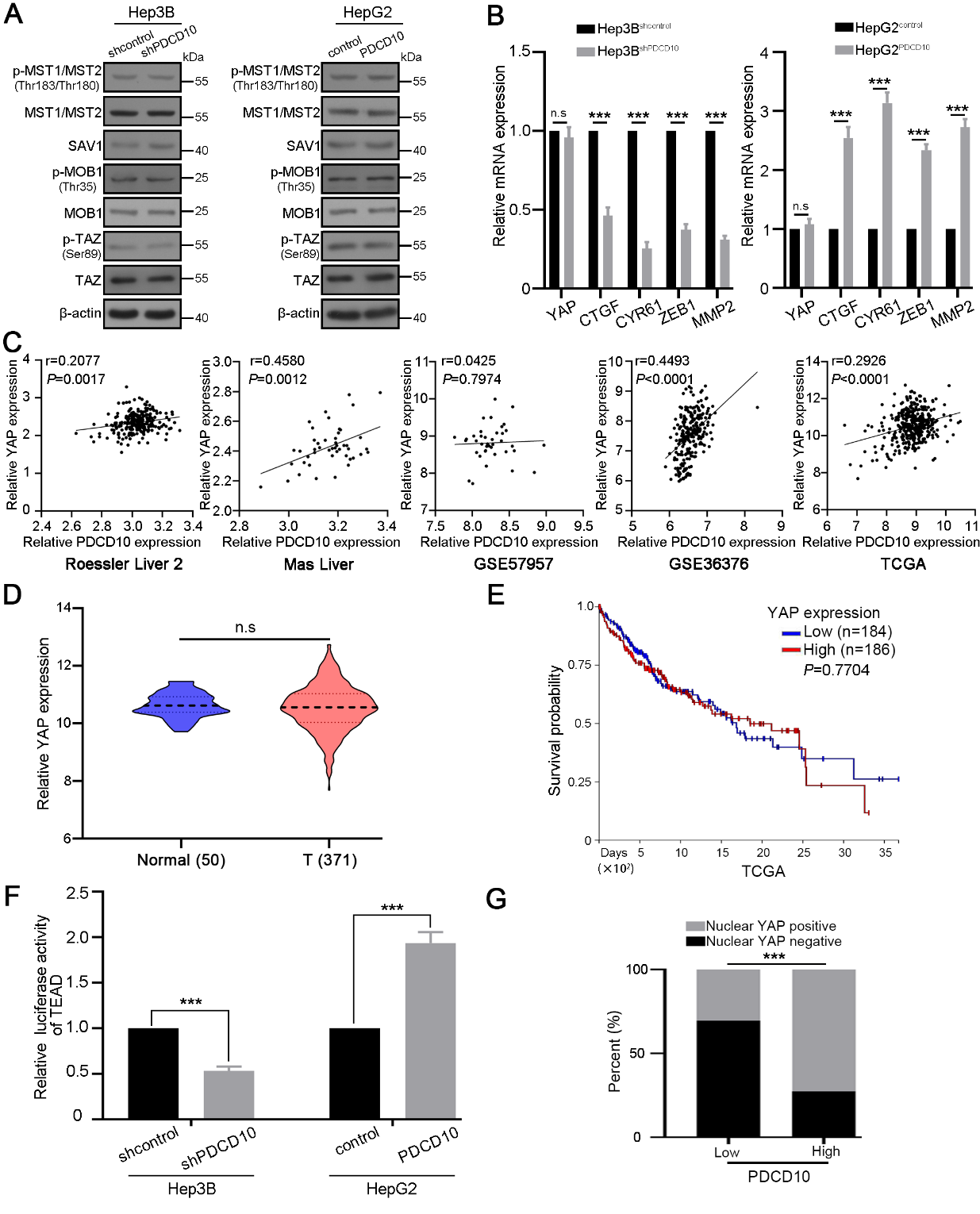


**Fig. S4 PDCD10 promotes YAP activation in HCC.** (A) Western blot analysis for key proteins of Hippo signaling in Hep3B^shPDCD10^, HepG2^PDCD10^ and their control cells. (B) qRT-PCR analysis of YAP and its representative downstream effectors in Hep3B^shPDCD10^, HepG2^PDCD10^ and their control cells. Data are the mean ± SD of three independent experiments. (C) Pearson correlation analysis of PDCD10 and YAP mRNA in Oncomine (Roessler Liver 2, Mas Liver), GEO (GSE57957, GSE57957), and TCGA datasets. (D) Comparing the level of YAP mRNA in HCC tissues and normal liver tissues from TCGA HCC dataset. (E) Kaplan-Meier analysis of survival probability for HCC patients based on YAP mRNA expression using public data from TCGA dataset. (F) Double luciferase reporter assay showed the TEAD transcriptional activity in Hep3B^shPDCD10^, HepG2^PDCD10^ and their control cells. HCC cells were transfected with TEAD luciferase reporter plasmid 8xGTIIC-luciferase as well as with pRL-CMV Renilla luciferase plasmid using Lipofectamine LTX. Data are the mean ± SD of three independent experiments. (G) Statistic analysis showed PDCD10 was positively correlated with nuclear YAP localization in overall cohort (training plus validation cohorts). ***, *P*<0.001.


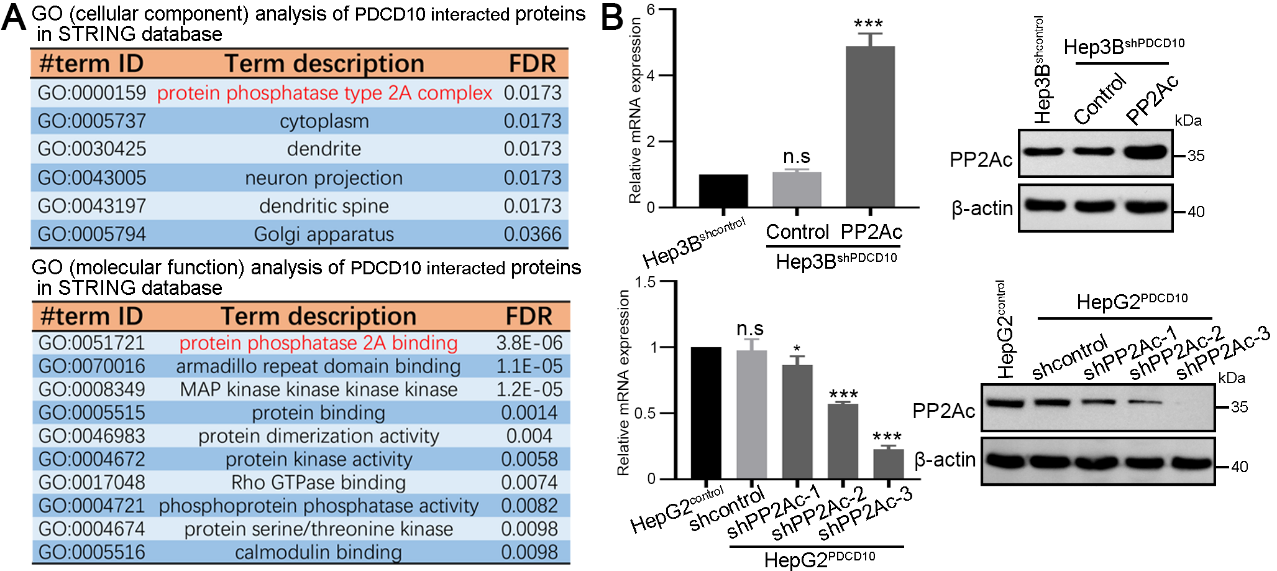


**Fig. S5 PDCD10 was associated with protein phosphatase type 2A (PP2A) complex and PP2A binding.** (A) GO functional enrichments of PDCD10 interacted proteins in STRING database showed PDCD10 was significantly associated with PP2A complex and PP2A binding. (B) The knockdown and overexpression efficacy of PP2Ac in indicated HCC cells were detected by qRT-PCR and western blot. n.s, no significance; *, *P*<0.05; ***, *P*<0.001.


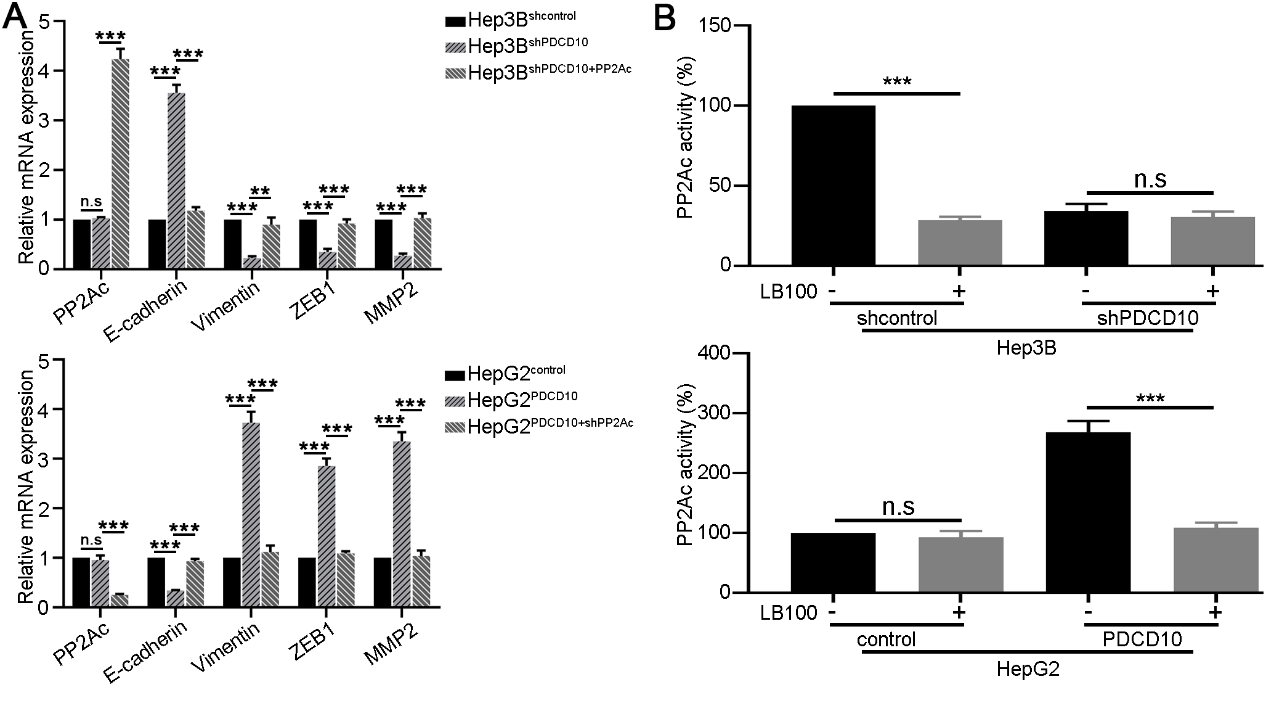


**Fig.S6 PP2Ac is critical for PDCD10 mediated progression of HCC.** (A) mRNA expression of EMT markers in Hep3B^shPDCD10^ cells with PP2Ac further overexpression and in HepG2^PDCD10^ cells with PP2Ac further knockdown. Data are the mean ± SD of three independent experiments. (B) PP2Ac activity was detected in indicated HCC cells with or without LB-100 treatment (10 μM for 48 hours). Data are the mean ± SD of three independent experiments. n.s, no significance; **, *P*<0.01; ***, *P*<0.001.
