# Supplementary material for: Programmed cell death 10 promotes metastasis and epithelial-mesenchymal transition of hepatocellular carcinoma via PP2Ac-mediated YAP activation
Source: Cell Death Dis. 2021 Sep 14;12(9):849. doi: 10.1038/s41419-021-04139-z (PMC8440642; doi:10.1038/s41419-021-04139-z)
Supplement: Supplementary file 2 — Supplementary Tables [file 41419_2021_4139_MOESM2_ESM.docx]

Table S1. Clinicopathological characteristics of HCC patients in training cohort and validation cohort

| **Clinicopathological Variables** | **Cases** | | P Value |
| --- | --- | --- | --- |
|  | **Training Cohort** | **Validation Cohort** |  |
| Gender |  |  |  |
| Female | 42 | 34 |  |
| Male | 118 | 106 | 0.696 |
| Age (years) |  |  |  |
| ≥60 | 102 | 85 |  |
| <60 | 58 | 55 | 0.588 |
| HBsAg |  |  |  |
| Negative | 24 | 23 |  |
| Positive | 136 | 117 | 0.734 |
| Liver cirrhosis |  |  |  |
| Absence | 48 | 40 |  |
| Presence | 112 | 100 | 0.786 |
| AFP |  |  |  |
| <20ng/L | 66 | 52 |  |
| ≥20ng/L | 94 | 88 | 0.468 |
| Tumor number |  |  |  |
| Solitary | 68 | 64 |  |
| Multiple | 92 | 76 | 0.576 |
| Tumor size |  |  |  |
| ≤5 cm | 51 | 52 |  |
| >5 cm | 109 | 88 | 0.338 |
| Microvascular invasion |  |  |  |
| Absence | 87 | 82 |  |
| Presence | 73 | 58 | 0.465 |
| Capsular formation |  |  |  |
| Presence | 86 | 65 |  |
| Absence | 74 | 75 | 0.206 |
| Child-Pugh |  |  |  |
| A | 120 | 96 |  |
| B | 40 | 44 | 0.216 |
| Edmondson-Steiner grade |  |  |  |
| I-II | 86 | 71 |  |
| III-IV | 74 | 69 | 0.599 |
| TNM stage |  |  |  |
| I- II | 65 | 58 |  |
| III-IV | 95 | 82 | 0.888 |
| BCLC stage |  |  |  |
| 0-A | 70 | 64 |  |
| B-C | 90 | 76 | 0.733 |

Table S2. Univariate and multivariate analysis of risk factors associated with OS and DFS of HCC patients in training cohort

| **Variables** | **OS** | | | | |  | **DFS** | | | | |
| --- | --- | --- | --- | --- | --- | --- | --- | --- | --- | --- | --- |
|  | **Univariate Analysis** | |  | **Multivariate Analysis** | |  | **Univariate Analysis** | |  | **Multivariate Analysis** | |
|  | **HR (95% CI)** | ***P*** |  | **HR (95% CI)** | ***P*** |  | **HR (95% CI)** | ***P*** |  | **HR (95% CI)** | ***P*** |
| Gender |  | | | | | | | | | | |
| Female | 1 |  |  |  |  |  | 1 |  |  |  |  |
| Male | 1.146(0.747-1.758) | 0.532 |  |  | NA |  | 1.258 (0.840-1.884) | 0.265 |  |  | NA |
| Age (years) |  | | | | | | | | | | |
| <60 | 1 |  |  |  |  |  | 1 |  |  |  |  |
| ≥60 | 1.326(0.907-1.941) | 0.146 |  |  | NA |  | 1.309 (0.919-1.865) | 0.135 |  |  | NA |
| HBsAg |  | | | | | | | | | | |
| Negative | 1 |  |  |  |  |  | 1 |  |  |  |  |
| Positive | 1.254(0.738-2.129) | 0.403 |  |  | NA |  | 1.469(0.928-2.324) | 0.101 |  |  | NA |
| Liver cirrhosis |  | | | | | | | | | | |
| Absence | 1 |  |  |  |  |  | 1 |  |  |  |  |
| Presence | 1.267(0.833-1.925) | 0.269 |  |  | NA |  | 1.285(0.875- 1.889) | 0.201 |  |  | NA |
| AFP |  | | | | | | | | | | |
| <20ng/L | 1 |  |  |  |  |  | 1 |  |  |  |  |
| ≥20ng/L | 1.455(0.995-2.127) | 0.053 |  |  | NA |  | 1.305(0.919-1.853) | 0.137 |  |  | NA |
| Tumor number |  | | | | | | | | | | |
| Solitary | 1 |  |  | 1 |  |  | 1 |  |  | 1 |  |
| Multiple | 1.788(1.210-2.640) | **0.004** |  | 1.388(0.916-2.103) | 0.122 |  | 1.480(1.038-2.109) | **0.030** |  | 1.098(0.750-1.608) | 0.631 |
| Tumor size |  | | | | | | | | | | |
| ≤5 cm | 1 |  |  |  |  |  | 1 |  |  |  |  |
| >5 cm | 1.408(0.929-2.133) | 0.106 |  |  | NA |  | 1.283(0.880-1.870) | 0.195 |  |  | NA |
| Microvascular invasion |  | | | | | | | | | | |
| Absence | 1 |  |  | 1 |  |  | 1 |  |  | 1 |  |
| Presence | 1.694(1.167-2.460) | **0.006** |  | 1.216(0.818-1.809) | 0.333 |  | 1.633(1.154-2.310) | **0.006** |  | 1.215(0.838-1.762) | 0.304 |
| Capsular formation |  | | | | | | | | | | |
| Presence | 1 |  |  |  |  |  | 1 |  |  |  |  |
| Absence | 1.324(0.909-1.927) | 0.144 |  |  | NA |  | 1.225(0.865-1.734) | 0.253 |  |  | NA |
| Child-Pugh |  | | | | | | | | | | |
| A | 1 |  |  |  |  |  | 1 |  |  |  |  |
| B | 1.223(0.796-1.877) | 0.358 |  |  | NA |  | 1.439(0.978-2.117) | 0.064 |  |  | NA |
| Edmondson Steiner grade |  | | | | | | | | | | |
| I-II | 1 |  |  | 1 |  |  | 1 |  |  | 1 |  |
| III-IV | 2.965(2.030-4.331) | **0.000** |  | 3.240(2.168-4.842) | **0.000** |  | 2.853(2.006-4.057) | **0.000** |  | 3.275(2.240-4.787) | **0.000** |
| TNM stage |  | | | | | | | | | | |
| I- II | 1 |  |  | 1 |  |  | 1 |  |  | 1 |  |
| III-IV | 1.912(1.292-2.830) | **0.001** |  | 1.589(1.048-2.409) | **0.029** |  | 2.039(1.413-2.941) | **0.000** |  | 1.665(1.126-2.461) | **0.011** |
| BCLC stage |  | | | | | | | | | | |
| 0-A | 1 |  |  | 1 |  |  | 1 |  |  | 1 |  |
| B-C | 1.854(1.264-2.720) | **0.002** |  | 1.597(1.064-2.398) | **0.024** |  | 1.628(1.146-2.313) | **0.006** |  | 1.520(1.041-2.220) | **0.030** |
| PDCD10 expression |  | | | | | | | | | | |
| Low | 1 |  |  | 1 |  |  | 1 |  |  | 1 |  |
| High | 1.977(1.314-2.974) | **0.001** |  | 1.828(1.145-2.916) | **0.011** |  | 2.047(1.396-3.001) | **0.000** |  | 2.075(1.334-3.228) | **0.001** |

Table S3. Univariate and multivariate analysis of risk factors associated with OS and DFS of HCC patients in validation cohort

| **Variables** | **OS** | | | | |  | **DFS** | | | | |
| --- | --- | --- | --- | --- | --- | --- | --- | --- | --- | --- | --- |
|  | **Univariate Analysis** | |  | **Multivariate Analysis** | |  | **Univariate Analysis** | |  | **Multivariate Analysis** | |
|  | **HR (95% CI)** | ***P*** |  | **HR (95% CI)** | ***P*** |  | **HR (95% CI)** | ***P*** |  | **HR (95% CI)** | ***P*** |
| Gender |  | | | | | | | | | | |
| Female | 1 |  |  |  |  |  | 1 |  |  |  |  |
| Male | 1.164(0.734-1.847) | 0.518 |  |  | NA |  | 1.227 (0.791-1.904) | 0.361 |  |  | NA |
| Age (years) |  | | | | | | | | | | |
| <60 | 1 |  |  |  |  |  | 1 |  |  |  |  |
| ≥60 | 1.152(0.767-1.729) | 0.495 |  |  | NA |  | 1.096 (0.748-1.605) | 0.638 |  |  | NA |
| HBsAg |  | | | | | | | | | | |
| Negative | 1 |  |  |  |  |  | 1 |  |  |  |  |
| Positive | 1.294(0.766-2.186) | 0.335 |  |  | NA |  | 1.144(0.691-1.896) | 0.601 |  |  | NA |
| Liver cirrhosis |  | | | | | | | | | | |
| Absence | 1 |  |  |  |  |  | 1 |  |  |  |  |
| Presence | 1.513(0.982-2.330) | 0.060 |  |  | NA |  | 1.331(0.886- 2.000) | 0.168 |  |  | NA |
| AFP |  | | | | | | | | | | |
| <20ng/L | 1 |  |  |  |  |  | 1 |  |  |  |  |
| ≥20ng/L | 1.468(0.961-2.245) | 0.076 |  |  | NA |  | 1.398(0.945-2.069) | 0.094 |  |  | NA |
| Tumor number |  | | | | | | | | | | |
| Solitary | 1 |  |  |  |  |  | 1 |  |  |  |  |
| Multiple | 1.248(0.834-1.869) | 0.281 |  |  | NA |  | 1.343(0.922-1.956) | 0.125 |  |  | NA |
| Tumor size |  | | | | | | | | | | |
| ≤5 cm | 1 |  |  |  |  |  | 1 |  |  | 1 |  |
| >5 cm | 1.380(0.908-2.096) | 0.131 |  |  | NA |  | 1.519(1.026-2.249) | **0.037** |  | 1.220(0.806-1.845) | 0.347 |
| Microvascular invasion |  | | | | | | | | | | |
| Absence | 1 |  |  | 1 |  |  | 1 |  |  | 1 |  |
| Presence | 2.154(1.441-3.222) | **0.000** |  | 1.663(1.072-2.579) | **0.023** |  | 2.041(1.396-2.984) | **0.000** |  | 1.724(1.142-2.604) | **0.010** |
| Capsular formation |  | | | | | | | | | | |
| Presence | 1 |  |  |  |  |  | 1 |  |  |  |  |
| Absence | 1.490(0.998-2.223) | 0.051 |  |  | NA |  | 1.295(0.891-1.881) | 0.175 |  |  | NA |
| Child-Pugh |  | | | | | | | | | | |
| A | 1 |  |  |  |  |  | 1 |  |  |  |  |
| B | 1.302(0.852-1.992) | 0.223 |  |  | NA |  | 1.149(0.770-1.716) | 0.497 |  |  | NA |
| Edmondson Steiner grade |  | | | | | | | | | | |
| I-II | 1 |  |  | 1 |  |  | 1 |  |  | 1 |  |
| III-IV | 1.641(1.099-4.451) | **0.016** |  | 1.037(0.673-1.598) | 0.869 |  | 1.570(1.082-2.279) | **0.018** |  | 1.155(0.776-1.718) | 0.479 |
| TNM stage |  | | | | | | | | | | |
| I- II | 1 |  |  | 1 |  |  | 1 |  |  | 1 |  |
| III-IV | 2.578(1.670-3.979) | **0.000** |  | 2.109(1.333-3.335) | **0.001** |  | 1.816(1.238-2.664) | **0.002** |  | 1.463(0.973-2.200) | 0.068 |
| BCLC stage |  | | | | | | | | | | |
| 0-A | 1 |  |  | 1 |  |  | 1 |  |  | 1 |  |
| B-C | 2.477(1.627-3.771) | **0.000** |  | 1.754(1.089-2.828) | **0.021** |  | 2.134(1.454-3.133) | **0.000** |  | 1.687(1.082-2.630) | **0.021** |
| PDCD10 expression |  | | | | | | | | | | |
| Low | 1 |  |  | 1 |  |  | 1 |  |  | 1 |  |
| High | 2.036(1.282-3.233) | **0.003** |  | 1.717(1.051-2.803) | **0.031** |  | 2.113(1.380-3.235) | **0.001** |  | 1.808(1.136-2.878) | **0.013** |

Table S4. The full list of PDCD10 interacting proteins in STRING database

| Node 1 | Node 2 | Node 2 annotation |
| --- | --- | --- |
| PDCD10 | PPP2R1B | Serine/threonine-protein phosphatase 2A 65 kDa regulatory subunit A beta isoform |
| PDCD10 | STK3 | Serine/threonine-protein kinase 3 |
| PDCD10 | PPP2CA | Serine/threonine-protein phosphatase 2A catalytic subunit alpha isoform |
| PDCD10 | CTTNBP2 | Cortactin-binding protein 2 |
| PDCD10 | PPP2R1A | Serine/threonine-protein phosphatase 2A 65 kDa regulatory subunit A alpha isoform |
| PDCD10 | SIKE1 | Suppressor of IKBKE 1 |
| PDCD10 | STRN3 | Striatin-3 |
| PDCD10 | STRN4 | Striatin-4 |
| PDCD10 | FGFR1OP2 | FGFR1 oncogene partner 2 |
| PDCD10 | PTPN13 | Tyrosine-protein phosphatase non-receptor type 13 |
| PDCD10 | SLMAP | Sarcolemmal membrane-associated protein |
| PDCD10 | STRIP1 | Striatin-interacting protein 1 |
| PDCD10 | STRN | Striatin |
| PDCD10 | KRIT1 | Krev interaction trapped protein 1 |
| PDCD10 | CTTNBP2NL | CTTNBP2 N-terminal-like protein |
| PDCD10 | MOB4 | MOB-like protein phocein |
| PDCD10 | STK24 | Serine/threonine-protein kinase 24 |
| PDCD10 | CCM2 | Cerebral cavernous malformations 2 protein |
| PDCD10 | MST4 | Serine/threonine-protein kinase 26 |
| PDCD10 | STK25 | Serine/threonine-protein kinase 25 |

Table S5. The sequences of PCR primers used in this study

| **Genes** | **Application** | **Sequence (5’→3’)** |
| --- | --- | --- |
| PDCD10 | qRT-PCR | F: GCCCCTCTATGCAGTCATGTA |
|  |  | R: AGCCTTGATGAAAGCGGCTC |
| GAPDH | qRT-PCR | F: ACAACTTTGGTATCGTGGAAGG |
|  |  | R: GCCATCACGCCACAGTTTC |
| E-cadherin | qRT-PCR | F: CGAGAGCTACACGTTCACGG |
|  |  | R: GGGTGTCGAGGGAAAAATAGG |
| Vimentin | qRT-PCR | F: AGTCCACTGAGTACCGGAGAC |
|  |  | R: CATTTCACGCATCTGGCGTTC |
| CTGF | qRT-PCR | F: ACCGACTGGAAGACACGTTTG |
|  |  | R: CCAGGTCAGCTTCGCAAGG |
| CYR61 | qRT-PCR | F: CGGCTCCCTGTTTTTGGAATG |
|  |  | R: GGGTTTCTTTCACAAGGCGG |
| ZEB1 | qRT-PCR | F: CAGCTTGATACCTGTGAATGGG |
|  |  | R: TATCTGTGGTCGTGTGGGACT |
| MMP2 | qRT-PCR | F: GATACCCCTTTGACGGTAAGGA |
|  |  | R: CCTTCTCCCAAGGTCCATAGC |
| PP2Ac | qRT-PCR | F: CAAAAGAATCCAACGTGCAAGAG |
|  |  | R: CGTTCACGGTAACGAACCTT |
| YAP | qRT-PCR | F: TAGCCCTGCGTAGCCAGTTA |
|  |  | R: TCATGCTTAGTCCACTGTCTGT |

Table S6. The primary antibodies used in this study

| **Primary Antibodies** | **Origin** | **Application** | **Dilution** |
| --- | --- | --- | --- |
| PDCD10 | Proteintech (10294-2-AP) | IHC | 1:50 |
|  |  | WB | 1:500 |
|  |  | IP | 1:300 |
|  |  | IF | 1:200 |
| E-cadherin | CST (#14472) | WB | 1:1000 |
|  |  | IF | 1:200 |
|  |  | IHC | 1:100 |
| Vimentin | CST (#5741) | WB | 1:1000 |
|  |  | IF | 1:200 |
|  |  | IHC | 1:100 |
| LATS1/2 | Affinity (DF7517) | WB | 1:1000 |
| p-LATS1/2  (Ser909/Ser872) | Affinity (AF8163) | WB | 1:1000 |
| YAP | CST (#14074) | WB | 1:1000 |
|  |  | IHC | 1:400 |
|  |  | IF | 1:100 |
| p-YAP  (Ser127) | CST (#13008) | WB | 1:1000 |
| p-MST1/MST2  (Thr183/Thr180) | CST (#49332) | WB | 1:1000 |
| MST1/MST2 | Affinity (DF8569) | WB | 1:1000 |
| SAV1 | CST (#13301) | WB | 1:1000 |
| p-MOB1 (Thr35) | CST (#8699) | WB | 1:1000 |
| MOB1 | CST (#13730) | WB | 1:1000 |
| p-TAZ (Ser89) | Affinity (AF4315) | WB | 1:1000 |
| TAZ | Affinity (DF4653) | WB | 1:1000 |
| CTGF | Affinity (DF7091) | WB | 1:1000 |
| CYR61 | Affinity (DF6250) | WB | 1:1000 |
| ZEB1 | Affinity (DF7414) | WB | 1:1000 |
| MMP2 | Affinity (AF0577) | WB | 1:1000 |
| PP2Ac | Santa Cruz (sc-80665) | WB | 1:500 |
|  |  | IP | 1:200 |
|  |  | IF | 1:100 |
| His Tag | ProteinTech (66005-1-Ig) | WB | 1:5000 |
| GST Tag | ProteinTech (10000-0-AP) | WB | 1:1000 |
| β-actin | ProteinTech (60008-1-Ig) | WB | 1:5000 |
| Histone H3 | Affinity (AF0863) | WB | 1:1000 |

Table S7. List of secondary antibodies and reagents used in this study

| **Secondary antibodies or reagents** | **Origin** | **Application** | **Dilution** |
| --- | --- | --- | --- |
| Goat anti-Rabbit IgG (H+L) Secondary Antibody, DyLight 594 conjugate | ThermoFisher Scientific (#35560) | IF | 1:100 |
| Goat anti-Rabbit IgG (H+L) Secondary Antibody, DyLight 488 conjugate | ThermoFisher Scientific (#35553) | IF | 1:100 |
| Goat anti-Mouse IgG (H+L) Secondary Antibody, DyLight 594 conjugate | ThermoFisher Scientific (#35510) | IF | 1:100 |
| Goat anti-Mouse IgG (H+L) Secondary Antibody, DyLight 488 conjugate | ThermoFisher Scientific (#35503) | IF | 1:100 |
| Goat anti-Mouse IgG(H+L)/HRP | ZSGB-BIO (ZB-2305) | WB | 1:4000 |
| Goat anti-Rabbit IgG(H+L)/HRP | ZSGB-BIO (ZB-2301) | WB | 1:4000 |
| Hematoxylin and eosin (H&E) staining kit | Solarbio | HE staining | / |

Table S8. The sequences of shRNAs used in this study

| Name | Sequence |
| --- | --- |
| PDCD10-shRNA-1 | 5’-CAGGATGTTGAATGGGATTAT-3’ |
| PDCD10-shRNA-2 | 5’-CGTAAGTGCCAACCGACTAAT-3’ |
| PDCD10-shRNA-3 | 5’-GCCCTCATAAGCTTTGCACAA-3’ |
| PP2Ac-shRNA-1 | 5’-TGGAACTTGACGATACTCTAA-3’ |
| PP2Ac-shRNA-2 | 5’-CCCATGTTGTTCTTTGTTATT-3’ |
| PP2Ac-shRNA-3 | 5’-ACCGGAATGTAGTAACGATTT-3’ |
